# Comprehensive transcriptome analysis reveals distinct regulatory programs during vernalization and floral bud development of orchardgrass (*Dactylis glomerata* L.)

**DOI:** 10.1186/s12870-017-1170-8

**Published:** 2017-11-22

**Authors:** Guangyan Feng, Linkai Huang, Ji Li, Jianping Wang, Lei Xu, Ling Pan, Xinxin Zhao, Xia Wang, Ting Huang, Xinquan Zhang

**Affiliations:** 1 Department of Grassland Science, College of Animal Science and Technology, Sichuan Agricultural University, Chengdu, China; 2 Agronomy Department, University of Florida, Gainesville, FL USA

**Keywords:** *Dactylis glomerata* L., Flowering regulation, High-throughput sequencing, Orchardgrass, Transcriptome, Vernalization

## Abstract

**Background:**

Vernalization and the transition from vegetative to reproductive growth involve multiple pathways, vital for controlling floral organ formation and flowering time. However, little transcription information is available about the mechanisms behind environmental adaption and growth regulation. Here, we used high-throughput sequencing to analyze the comprehensive transcriptome of *Dactylis glomerata* L. during six different growth periods.

**Results:**

During vernalization, 4689 differentially expressed genes (DEGs) significantly increased in abundance, while 3841 decreased. Furthermore, 12,967 DEGs were identified during booting stage and flowering stage, including 7750 up-regulated and 5219 down-regulated DEGs. Pathway analysis indicated that transcripts related to circadian rhythm, photoperiod, photosynthesis, flavonoid biosynthesis, starch, and sucrose metabolism changed significantly at different stages. Coexpression and weighted correlation network analysis (WGCNA) analysis linked different stages to transcriptional changes and provided evidence of inner relation modules associated with signal transduction, stress responses, cell division, and hormonal transport.

**Conclusions:**

We found enrichment in transcription factors (TFs) related to *WRKY, NAC, AP2/EREBP, AUX/IAA, MADS-BOX, ABI3/VP1, bHLH,* and the *CCAAT* family during vernalization and floral bud development. TFs expression patterns revealed intricate temporal variations, suggesting relatively separate regulatory programs of TF modules. Further study will unlock insights into the ability of the circadian rhythm and photoperiod to regulate vernalization and flowering time in perennial grass.

**Electronic supplementary material:**

The online version of this article (10.1186/s12870-017-1170-8) contains supplementary material, which is available to authorized users.

## Background

Flowering is a critical developmental stage of most higher plants, which makes the plants produce seeds, thus to pass the genetic information from one to the next generation. The timing of flowering is regulated by multiple genetic and environmental factors. Specifically, the plant genes controlling flowering are induced via synchronization of climatic and environmental conditions. Thus, flowering time varies extensively according to climate and latitudinal or altitudinal gradients. For crop production, it is important to coordinate the flowering time with changes of environment to avoiding adverse natural conditions during flower differentiation [1]. With the advance of molecular biology, genes controlling flowering time in annual crops such as *Oryza sativa, Triticum aestivum, Hordeum vulgare*, and especially in model plants *Arabidopsis thaliana*, have been identified, which mainly belong to four pathways interacting to each other including photoperiod pathway [2], GA pathway [3], vernalization pathway, and autonomous pathway [1, 4]. For winter cereals, low temperatures and long days following low temperatures are the major driving factors for plant development and flowering.

Vernalization or jarovization was first defined by Lysenko et al. in 1986 when it was observed that wheat varieties required cold for stem elongation and flowering [5]. Vernalization in winter cereals depends on a highly integrated and complex system of development, involving numerous structural and regulatory genes in an intricate network of signaling pathways. Flowering time of certain species strongly correlated with day time length and temperature during vernalization [6]. In wheat, chromosomes 5A and 5D play an important role in determining the responses to vernalization [7]. In barley, a series of quantitative trait loci (QTLs) in clusters associated with heading dates have been found on chromosome 7 [8]. Subsequent studies revealed other colinear regions of chromosomes 5A [9], 5B [10], and 5D in wheat; the genes were cloned in sequential studies and named the vernalization family (*VRN*) since they are extensively involved in the vernalization response. The latter demonstrated that *VRN3* is completely linked to a gene similar to the *FLOWERING LOCUS T (FT)* in *A.thaliana* [11]. A model was further developed to elaborate the function of *VRN1, VRN2,* and the FT genes in response to vernalization. Prior to cold exposure, *VRN2* represses the expression of *FT*. During cold exposure, *VRN1* expression increases, resulting in the repression of *VRN2,* which in turn allows activation of FT during long days, thus inducing flowering [12]. In *A. thaliana*, the vernalization pathway converge on *FLOWERING LOCUS C (FLC)*, a MAD-box transcription regulator; furthermore, its activator FRIGIDA represses flowering via increase of *FLC* mRNA abundance [13, 14]. The upstream gene *WRKY34*-induced and CULLIN3A (*CUL3A*)-dependent on FRIGIDA, modulate flowering in response to vernalization [15]. A latest study on transcriptome sequencing and chromatin immunoprecipitation sequencing (ChIP-seq) suggested that *VRN1* binds the promoter of *FLOWERING LOCUS T-like*, and also targets *VRN2* and *ODDSOC2* [16]. This reasearch provide more information on flowering regulation of cereal crop.

With the known genetic background and natural variations, *A. thaliana* is an excellent tool in studying the complexity of vernalization regulation. Currently, the full flowering network can only be approached in *A. thaliana*. Studies on *Oryza sativa, Triticum aestivum,* and *Hordeum vulgare* have led to the identification of components within individual signaling pathways that affect flowering as well as their positioning within molecular hierarchies. Even more importantly, the current study concentrates on annuals. In contrast to annuals, perennials require a long vegetative phase to accumulate and achieve the transition to the reproductive stage [17]. In addition, perennials still retain meristems in a vegetative state once they become adult [18]. Obvious differences and more complex molecular mechanisms are involved in vernalization, due to the differential life-cycles.

Orchardgrass (*Dactylis glomerata* L.) is a winter perennial gramineae grass, which is native to northern Africa, western and central Europe, and temperate Asia [19]. As an economically important perennial forage crop, orchardgrass has been widely used for a long time due to its good adaptability, abundant nutrients, and high biomass production [20]. Vernalization is an important physiological characteristic of orchardgrass, which decide the timing of flowering. A clear understanding of the molecular mechanism of flowering can provide a basis for targeted forage breeding. However, the molecular mechanisms that regulate vernalization of perennial grass species remain unclear. In this study, we generated a comprehensive and continuous transcriptome of orchardgrass to elaborate dynamic changes of gene expression among different stages and constructed the co-expression network of genes responding to vernalization and flowering regulating. This research will facilitate the understanding of flowering mechanisms of perennial grasses in general.

## Methods

### Plant material and RNA extraction

A orchardgrass variety DONATA (Registered No. 398) was grown in a greenhouse of the Sichuan Agricultural University located in Chengdu (30°42′N, 103°51′E; Chengdu city, Sichuan, China) under natural light conditions, then was transferred to field in Chongzhou farm (30°37′N, 103°40′; Chongzhou city, Sichuan, China) prior to temperature reduction in the year of 2016. For sampling, mixed young leaf samples were collected from three biological replicates at six different stages and immediately frozen in liquid nitrogen and stored in −80 °C freezers before being used. The six sampling stages included before vernalization January 4th, vernalization February 2nd, after vernalization March 2nd, vegetative growth stage March 24th, before heading April 9th, and heading stage May 5th. We defined these six sampling stages as before vernalization stage (BV_DON), vernalization stage (V_DON), after vernalization stage (AV_DON), vegetative growth stage (VG_DON), before heading stage (BH_DON), and heading stage (H_DON) using a sampling timeline (Additional file 1: Figure S1). From BV_DON to V_DON, this was defined as phase 1 (P1), V_DON to AV_DON was defined as phase 2 (P2), AV_DON to VG_DON was defined as phase 3 (P3), VG_DON to BH_DON was defined as phase 4 (P4), and BH_DON to H_DON was defined as phase 5 (P5) in our data analysis. DON refers to the orchardgrass cultivated variety DONATA.

Total RNA was extracted from samples by using the RNAprep pure plant kit (Tiangen Biochemical Technology Company, Beijing, China). RNA samples were treated with DNaseIto remove DNA, then RNA samples were monitored on 1% agarose gels. RNA purity was verified using the NanoPhotometer® spectrophotometer (IMPLEN, CA,USA) and the concentration was measured via the Qubit® RNA Assay Kit in Qubit® 2.0 Fluorimeter (Life Technologies, CA, USA). RNA integrity was assessed using the RNA Nano 6000 Assay Kit of the Agilent Bioanalyzer 2100 system (Agilent Technologies, CA, USA). RNA samples with 260/280 ratio between 1.8 and 2.0, 260/230 ratio between 2.0 and 2.5, and RNA integrity number above 8.0, were chosen for further library construction and sequencing.

### Transcriptome sequencing and data analysis

Approximately, 3 μg RNA for each sample was used for the RNA sample preparations and a total of eighteen sequencing libraries were generated using NEBNext® Ultra™ Directional RNA Library Prep Kit for Illumina® (NEB, USA) following manufacturer’s recommendations. Library construction and transcriptome sequencing were conducted by the Novogene Bioinformatics Institute (Beijing, China) on Illumina Hiseq 4000 platform (Illumina, San Diego, CA, USA). Without reference genome, the clean reads were assembled as a reference genome via Trinity software, according to standard parameters for next analysis. Read counts per gene were expressed as the expected number of Fragments Per Kilobase of transcript sequence per Million base pairs sequenced (FPKM). To depict the global abundance of gene expression, the FPKM values of transcripts were accessed via box-plot. All the assembled unigenes were searched and annotated against the publicly available protein databases including Nr, Nt, Pfam, KOG/COG(EuKaryotic Orthologous Groups/ Clusters of Orthologous Groups, Swiss-prot, KEGG(Kyoto Encyclopedia of Genes and Genomes), and GO (Gene Ontology), using BLASTx analysis with an E-value cut-off of 1.0E-05.

DEGs were identified via pairwise sample comparisons in adjacent sampling points (V_DON-BV_DON, AV_DON-V_DON, VG_DON-AV_DON, BH_DON-VG_DON and H_DON-BH_DON) by using DESeq R package (1.18.0) [21]. The expression levels were identified as significant DEGs by applying the cutoff at a fold change greater than 2 and *p*-value below 0.05(FDR *P* value < 0.01) [22].

To further and systematically predict the complex biological functions of genes and to identify active biological pathways among developmental phases, the assembled unigenes were mapped against both GO and KEGG databases. Genes with an adjusted *P*-value below 0.05 found by DESeq were assigned as differentially expressed and employed for GO and KEGG analyses. GO enrichment analysis of DEGs was implemented with the GOseq R package, in which the gene length bias was corrected. GO terms with corrected *P*-values below 0.05 were considered significantly enriched by DEGs [23]. KEGG enrichment analysis of DEGs in KEGG database (http://www.genome.jp/kegg/) and KOBAS software was used to test the statistical enrichment of DEGs in KEGG pathways [24].

The WGCNA package was used for a weighted gene coexpression network analysis in R (v3.3.0) as described by Langfelder et al. [25]. A total of 25,071 genes with FPKM values above 0 in more than two sampling points were used. A gene expression adjacency matrix was constructed for analyzing network topology, the soft thresholding power = 7 in our analysis. The blockwiseModules was used to obtaining the modules by default setting. A topological overlap matrix was calculated by comparing the connectivity similarities of each pair of probes among all genes. An eigen-gene network was constructed to represent the relationships among the modules. The networks were visualized using Cytoscape v.3.0.0.

Principal component analysis (PCA) and hierarchical clustering were performed to assess transcriptome similarity among samples and to evaluate sampling between biological replicates. PCA was performed based on expressed genes in different samples by using the R program with the default parameter. Hierarchical clustering of samples via the complete linkage method showed the change of gene expression levels across different stages. Self-organizing map (SOM) neural network was used for clustering analysis in search of co-expression DEGs groups [26]. Both of these clustering analysis were performed using the R language (v3.3.0). The heat map was obtained, using the OmicShare tools, which is a free online platform (www.omicshare.com/tools).

Unigenes were examined from the iTAK database for families of transcription factors or regulatory motifs (http://bioinfo.bti.cornell.edu/cgi-bin/itak/index.cgi) [27, 28].The local BLASTx similarity search was performed at E-value below 1.0E-05 against flowering-related genes sequences which were downloaded from NCBI database [29].

### Verification by qRT-PCR

The validity of RNA-seq were verified by quantitative real-time PCR(qRT-PCR). Eighteen flowering related genes of interest were validated using qRT-PCR, including *FRI* (c149523_g1), *LHP1* (c143664_g4), *VRN1*(c147469_g1), *VIP1*(c137956_g2), *LHY* (c146679_g3), *COL1* (c151793_g1), *WNK1* (c148831_g2), *CDF2* (c127155_g1), *GI* (c151406_g1), *COL16*(c140653_g3), *CRY1* (c137241_g1), *FD* (c128431_g1), *GAI* (c140748_g1), *FVE* (c137063_g1), *SPL* (c131707_g1), *SPL3* (c134262_g2), *SPL9* (c150483_g1) and *FLC-*like (c147268_g1). First-strand cDNAs were synthesized using Prime Script™ RT Master Mix Kit (RR036A) (Takara, Japan). The qRT-PCR reaction was performed using Bio-Rad CFX96 following the instructions for the SsoFast™ EvaGreen® Supermix Kit (SYBR Green) (#1725200AP) (Bio-Rad, USA). Three biological replicates were sampled were performed on each sample. The primers for unigenes were designed using online Primer BLAST program(https://www.ncbi.nlm.nih.gov/tools/primer-blast/index.cgi?LINK_LOC=BlastHome). Glyceraldehyde 3-phosphate dehydrogenase (*GAPDH*) was selected as reference gene [30]. Relative quantitative data were calculated based on the ΔΔCT method: normalization: (ΔCT = CT (sample) – CT (GAPDH); ΔΔCT = ΔCT (samples) - ΔCT (sample1); relative quantification = 2^-ΔΔCT^ [31]. All primers were listed in Additional file 2.

### Microscopic examination

Anatomic and microscopic examinations were conducted, using an Olymus CX41 microscope to distinguish the different stages and to accurately identify the floral initiation and development. The tissue was fixed with 75% (*v*/v) anhydrous ethanol and 25% (v/v) glacial acetic acid prior to microscopic examination. Pictures were obtained with an Olymus SZX12 stereo-microscope system (Olymus, Japan).

## Result

### Identification of morphological characteristics and RNA-seq data statistics

**Fig. 1:**
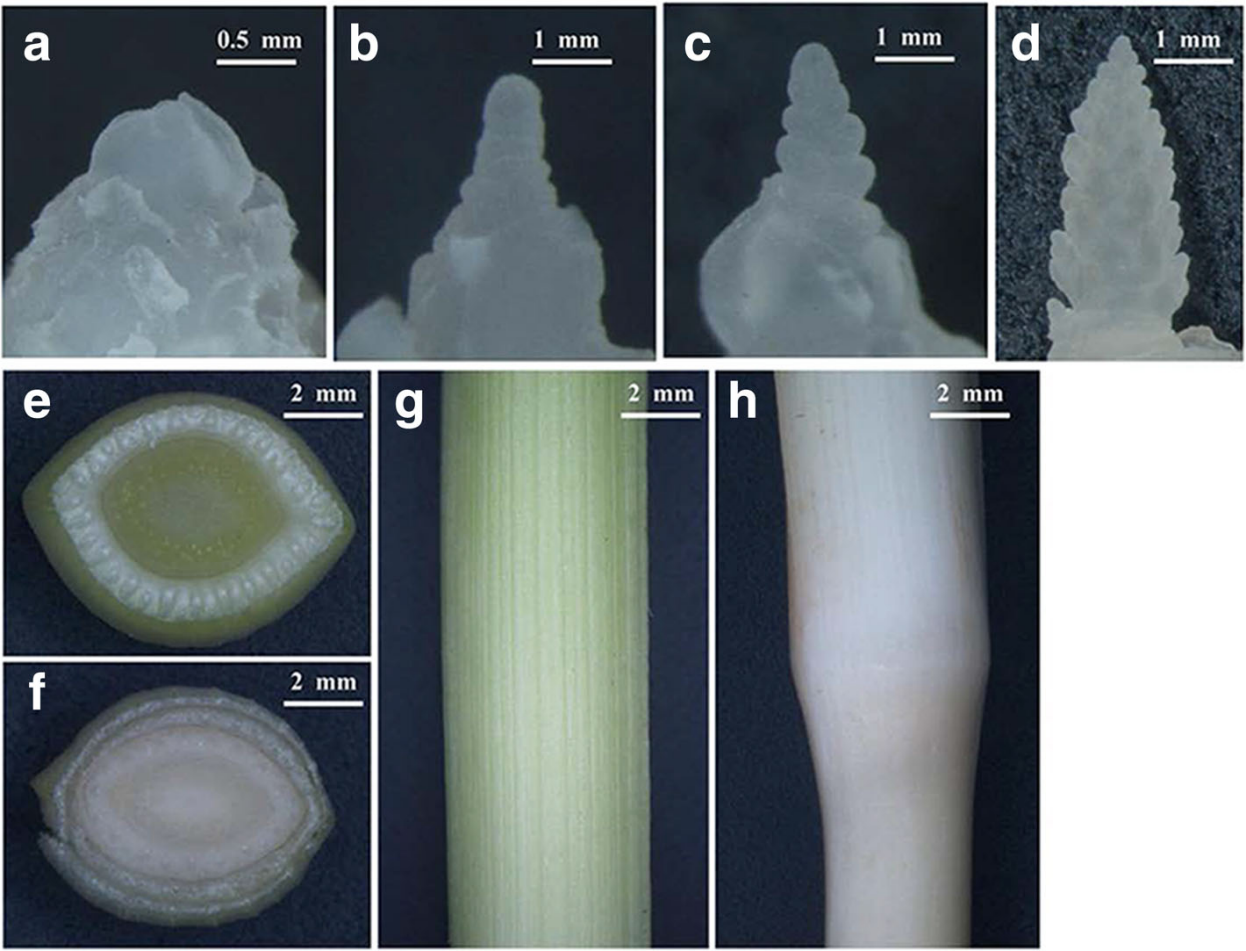
The morphological photographs of young inflorescence and stem. **a** Photographs of flower primordium after vernalization. **b**, **c** and **d**, the young inflorescence after flower primordium initiation for (**b**) 5 days (**c**) 10 days and (**d**) 3 weeks. **e** and **g**, Photographs of (**g**) stem and (**e**) transection of stem before young inflorescence formation. **f** and **h**, Photographs of (**h**) internodes and (**f**) transection of internodes after young inflorescence formation

We collected the samples weekly from December 2015 to May 2016 and determined the developmental stages via morphological and microscopic examination. The distinct stages were identified by changes of floral bud size (Fig. 1a, b, c, and d). Considering the development stage and climatic conditions, we identified six representative stages covering low temperature induction, booting stage, and flowering stage. In the first two phases (BV_DON and V_DON), orchardgrass plants have thickened lower internodes that form a crown (Additional file 3: Figure S2). The shoot apex and short stem are enclosed within the whorl of older leaf sheaths and near the ground level. After the flowering stimulus (AV_DON and VG_DON), the elongated stems (culms) of orchardgrass are divided into distinct nodes and hollows, but may be pithy or solid internodes, and these structures differentiate from the inflorescence (Fig. 1e, f, g, and h). The booting stage is critical for inflorescence formation. This stage was recognized by initiation and differentiation of floral organs from the flower primordium at the top of the reproductive stem.

To obtain an overview of the *Dactylis glomerata* L*.* transcriptome information during vernalization and at the floral stage, we generated a set of consecutive and comprehensive transcriptome. Three biological replicates at each sampling point, a total of 18 samples, were collected for RNA-seq. A total of 1,108,545,336 raw reads were generated (Additional file 4: Table S1) and were deposited in the National Center for Biotechnology Information in Sequence Read Archive with accession number of SRR5341102. After trimming, high-quality clean reads were retrieved ranged from 48 M to 69 M for each sample. The clean reads were assembled into a total of 499,624 transcripts (> 200 bp) with a N50 of 1356 bp and a N90 of 339 bp. The assembled transcript dataset included 270,221 sequences with a N50 of 1000 bp and a N90 of 263 bp (Additional file 5: Figure S3A and B). In total, 133,371 unigenes were annotated in at least one of these databases, 13,701 unigenes in all databases (Additional file 6: Table S2). The expression of unigenes were showed in Additional file 7: Figure S4.

### Identification of DEGs among stage comparisons

**Fig. 2:**
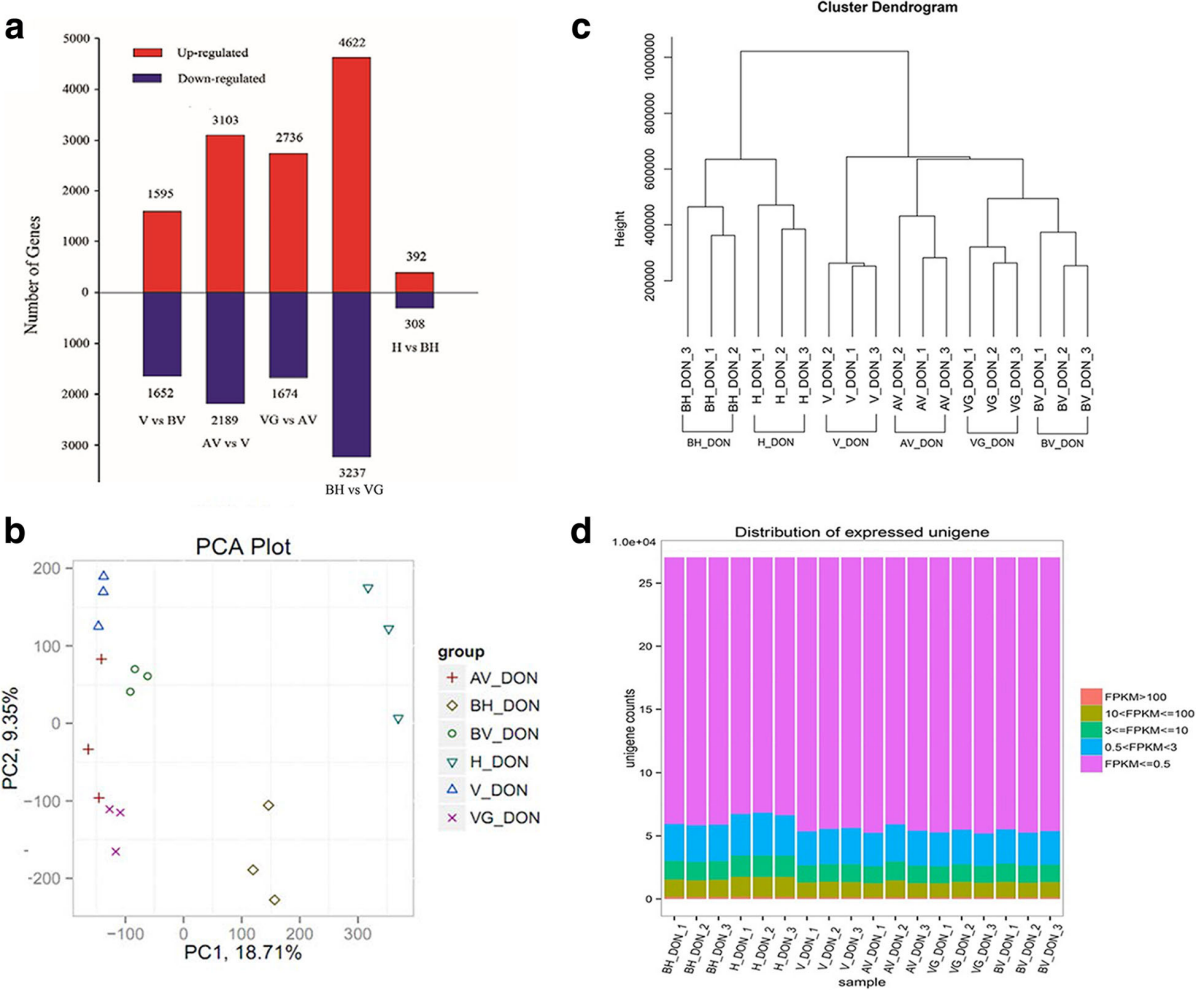
Transcriptional relationship between samples. **a** Number of up- and down-regulated genes in five pairwise sampling stages, including stage V_DON vs stage BV_DON (V vs BV), stage AV_DON vs stage V_DON (AV vs V), stage VG_DON vs stage AV_DON (VG vs AV), stage BH_DON vs stage VG_DON (BH vs VG), and stage H_DON vs stage BH_DON (H vs BH). **b** Principal component analysis based on all expressed genes, showing six distinct groups of samples: BV_DON_rep1, BV_DON_rep2, BV_DON_rep3 (light green circle); V_DON_rep1, V_DON_rep2, V_DON_rep3 (*blue regular triangle*); AV_DON_rep1, AV_DON_rep2, AV_DON_rep3 (*brown cross*); VG_DON_rep1, VG_DON_rep2, VG_DON_rep3 (*purple cross*); BH_DON_rep1, BH_DON_rep2, BH_DON_rep3 (*khaki rhombus*), H_DON_rep1, H_DON_rep2, H_DON_rep3 (*green inverted triangle*). **c** Cluster dendrogram, showing the global relationship between biological replicates. The y-axis shows the degree of variance. **d** Number of expressed unigenes and respective expression levels in each sample type, based on the FPKM of biological replicates. Sample labels are as follows: BV, before vernalization; V, vernalization; AV, after vernalization; VG, vegetative growth; BH, before heading; H, heading. DON refers to the orchardgrass cultivated varity DONATA (Registered No.398)

To investigate molecular differences among developmental stages, 21,508 DEGs were found in five pairwise stage comparisons, including 3247 in V_DON-BV_DON (V vs BV), 5292 in AV_DON-V_DON (AV vs V), 4410 in VG_DON-AV_DON (VG vs AV), 7859 in BH_DON-VG_DON (BH vs VG), and 700 in H_DON-BH_DON (H vs BH), respectively. Moreover, the number of up−/down-regulated genes were also presented (Fig. 2a). The number of DEGs in BH vs VG was remarkably higher than other pairwise comparisons, indicating the involvement of complex developmental procedures between stages VG_DON and BH_DON. The PCA analysis and hierarchical clustering revealed that the 18 samples clustered in six corresponding discrete groups. The sampling groups BV_DON and V_DON as well as AV_DON and VG_DON revealed a shorter distance than other stages, which was also showed in hierarchical clustering (Fig. 2b and c). The FPKM values of all 18 samples were accessed via box plots (Fig. 2d). These data constituted a consecutive and distinct set, highlighting the specialized nature of their transcriptomes related to their biological characteristics.

The GO and KEGG analysis indicated, the pathways of plant hormone signal transduction, circadian rhythm-plant, and flavonoid biosynthesis were significantly enriched in P1, relevant to hormone signal, cold response, light signaling, and vernalization induction in up/down-regulated DEGs. After more than a month of vernalization, the cold induction was disappeared as temperatures rose up during P2. The pathways of starch and sucrose metabolism were significantly enriched both in up or down-regulated DEGs. Furthermore, genes related to phenylpropanoid biosynthesis expressed abundantly in the group of up-regulated DEGs, and the result was consistent with morphological observations and gene expression profiling (Additional file 8: Figure S5A, B and Additional file 9: Figure S6A, B).

The transition of vegetative growth to reproductive growth occurred at P3. Large amounts of DEGs were identified involving in cellular biosynthetic process, intracellular component biosynthesis, macromolecule biosynthetic, and many other processes related to cell proliferation and differentiation (Additional file 8: Figure S5-C and Additional file 9: Figure S6-C). The floral primordia mainly generated and differentiated at P4; the DEGs within the pathways of starch and sucrose metabolism, ribosome and phenylpropanoid biosynthesis were presented at this stage. In contrast, the DEGs related to the pathways of circadian rhythm-plant, carbon metabolism, photosynthesis-antenna proteins, and biosynthesis of amino acids were down-regulated (Additional file 8: Figure S5-D and Additional file 9: Figure S6-D). The inflorescence protruded from the leaf sheath at P5, and the foremost biological process during this stage was pollen formation. The pathways of cell cycle, meiosis, DNA replication, mismatch repair, nucleotide excision repair, purine metabolism, and pyrimidine metabolism, which related to chromosome replication and meiosis were significantly enriched in up-regulated DEGs (Additional file 8: Figure S5-E and Additional file 9: Figure S6-E). These data suggest that specific regulators were required for different developmental phases, which converged to orchestrate the development of whole vernalization induction, floral bud formation, and heading.

### Temporal gene expression dynamics

**Fig. 3:**
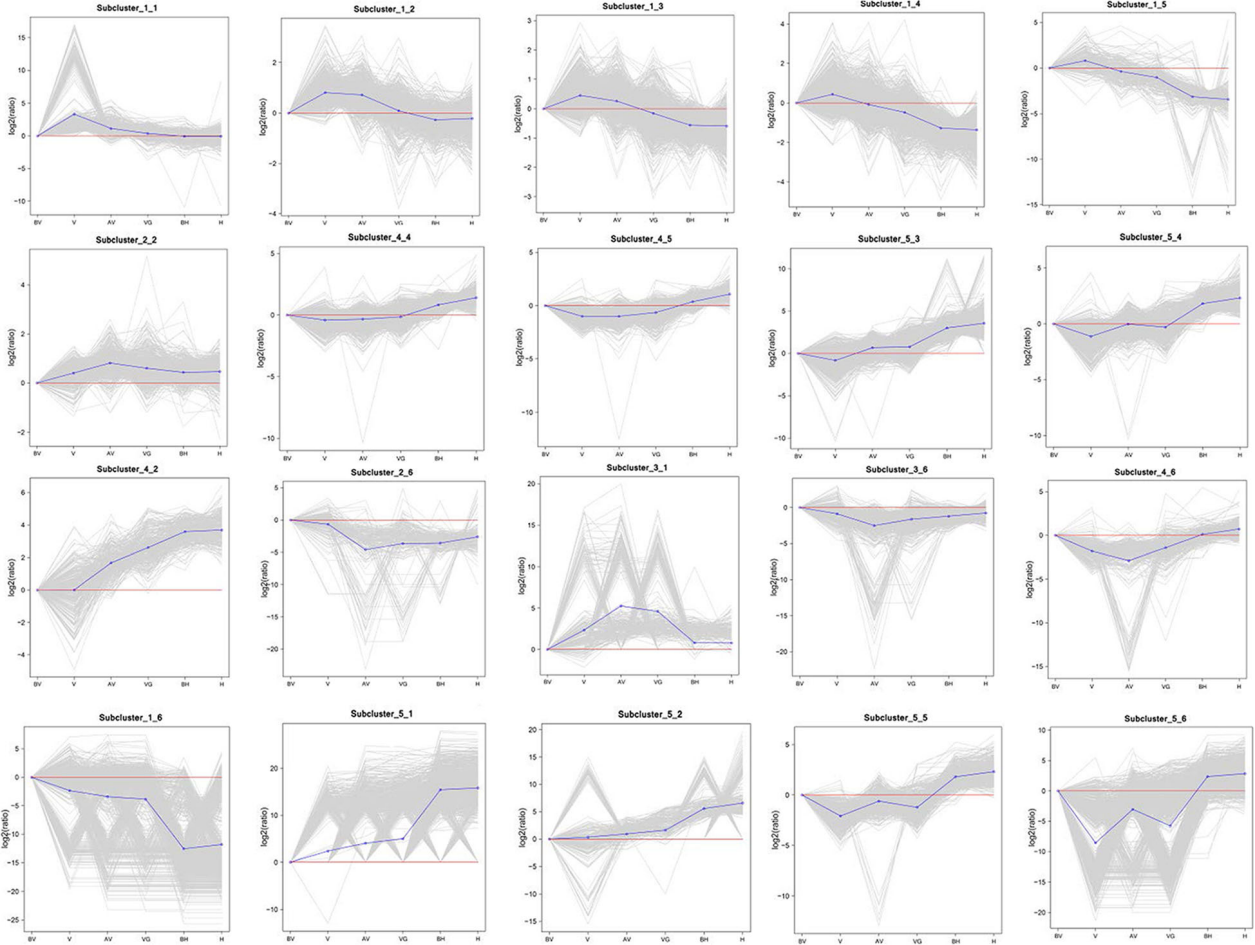
Clusters expression associated with stages. The x-axis shows the sampling stages, y-axis shows the relative expression.Sample labels are as follows: BV, before vernalization; V, vernalization; AV, after vernalization; VG, vegetative growth; BH, before heading; H, heading

To identify the major transcriptional dynamics associated with low temperature induced vernalization and the transition from vegetative growth to reproductive growth, the SOM-clustering approach were used to group genes according to similar expression profiles. A total of 6 coexpression clusters with 30 subclusters were defined (Additional file 10: Data S1. SOM-clustering results). Most subclusters showed a distinct expression peak at these 6 sampling points; however, the subcluster_5_5 and subcluster_5_6 were exceptional with two peaks. With the environment factors and growth situation within the gene expression dynamics, these sub-clusters were chosen and were looked into further (Fig. 3).

Supercluster 1 contained subcluster_1_1, subcluster_1_2, subcluster_1_3, subcluster_1_4, and subcluster_1_5, which comprised genes primarily expressed in phase V_DON with maximum expression. Subcluster_1_1 had overrepresentation of genes from processes associated to transport, regulation of transcription, metabolic processes, lipid and fatty biosynthetic processes, and signal transduction. The signal transduction categories contained many genes involved in protein phosphorylation, oxidation-reduction process response to cold, oxidative stress, and other abiotic stress. Genes response to light stimulus and photosynthesis were also identified in a variety of biosynthetic pathways; furthermore, several of the genes in circadian regulation of gene expression were found in this cluster, such as *CCA1* [32].

Subcluster_1_2 had an overrepresentation function category of ATP/ADP binding, protein binding, oxidative phosphorylation, and transcription factor activity mostly involved in DNA-templated transcription with the energy-related metabolism. Large numbers of DEGs in subcluster_1_3 were significantly enriched in plant hormone signal transduction, photosynthesis-antenna proteins, and circadian rhythm-plant pathways. Unigenes for hormone like auxin and ethylene were present in this cluster for functional action, including AUXIN RESPONSE FACTOR 18 *(ARF18)* and AUXIN RESPONSE FACTOR 22 *(ARF22)*, which encode transcription factors involved in auxin signaling. In addition, the auxin responsive protein INDOLE 3 ACETIC ACID 16 (*IAA16*), *IAA18*, *IAA28*, ethylene responsive transcription factor 061*(ERF061)* and *ERF073* were also found in this cluster.. Compared to subcluster_1_2, the pathways of photosynthesis and plant hormone signal transduction enriched more significantly in this cluster, as a response to light stimulus and abiotic stress.

For subcluster_1_4, a large proportion of genes in the sucrose metabolic process, starch metabolic process, and lipid metabolic process were linked. Many pathways such as cytoskeleton organization, cell wall macromolecule catabolic process, and acyl-carrier-protein biosynthetic process were involved in cell replication. In this cluster, several genes associated with calcium ion binding were expressed, which constituted part of the calcium signaling system involved in both the photosystem and late embryogenesis. Subcluster_1_5 comprised genes that expressed in molecular function of ATP binding, zinc ion binding, and DNA binding were involved in transferase activity, protein kinase activity, oxidoreductase activity, and hydrolase activity. The minimum expression peak of subcluster_4_2, subcluster_4_4, subcluster_4_5, subcluster_5_3, and subcluster_5_4 were also appeared in phase V_DON. After this time point, the gene expression demonstrates an increasing tendency in the subsequent developmental phases. These clusters including unigenes category are similar to supercluster 1, but have a completely opposite expression tendency in phase V_DON.

Subcluster_2_2, subcluster_2_6, subcluster_3_1, subcluster_3_6, and subcluster_4_6 showed an expression peak in phase AV_DON, but relatively low expression during other developmental phases. Genes in these clusters encode proteins with similar functions in signal transduction, protein phosphorylation, photosynthesis, oxidation-reduction process, and defense responses. While investigating the profile in subcluster_2_2, we identified several genes, encoding photosystem II reaction centre N protein (*psbN*). In subcluster_2_6 and subcluster_3_6, the genes showed the minimum expression in biological pathways such as oxidation-reduction processes. The genes of oxidation-reduction process, transport, sucrose metabolic process, carbohydrate metabolic process, and starch metabolic process were greatly changed relative to phase V_DON. Genes involved in cell morphogenesis and embryonic morphogenesis had minimal expression levels in subcluster_4_6 and genes response to auxin, reduction process, and nitrogen compound metabolic process had a maximum fold change compared to phase V_DON.

Prior to the heading phase, we focused on subcluster_1_6, subcluster_5_1, subcluster_5_2, subcluster_5_3, and subcluster_5_4, which all showed drastic expression changes. 290 genes in subcluster_5_2 followed an ascending expression trend overall, but with three expression peaks in both phases V_DON and BH_DON. Interestingly, the genes in V_DON showed an apparent polarization. For example, the expression transcription factor *bHLH35* in protein dimerization activity, and the AP2 domain for transcription factor activity sharply decreased at this stage. Several genes started to express and some other genes had maximum expression. We found many TFs, such as *bHLH35*, *bHLH92*, *BIM2*, and WRKY transcription factor 40 (*WRKY40*) and *WRKY70*. Ethylene-responsive transcription factor 2(*ERF2*) and the MADS-box transcription factor 34 were also highly expressed in stage BH_DON.

In subcluster_1_6, the genes relevant to stress responses, cell proliferation, morphological development, and iron uptake were detected,such as sigma-70(σ^70^) and sigma-54(σ^54^). Photomorphogenesis and photosystem regulated genes from the cytochrome family were also found in abundance in this cluster, including cytochrome P450, cytochrome oxidase C, cytochrome B6-F, and cytochrome b562.. In addition, subcluster_5_1 also expressed several genes of the cytochrome family, encoding cytochrome b and cytochrome b245.

Subcluster_5_1 and subcluster_5_2 showed peak expression in phase BH_DON with an increasing tendency from phase BV_DON. Functional categories of unique genes associated with genes encoding heat shock protein (*Hsp*) responding to stress were *Hsp* 70, *Hsp* 71, *Hsp* 83, *Hsp* 90, and *Hsp* 98 which were also presented in this cluster, as well as the identified salt stress-induced protein. In subcluster_1_6, several genes in abiotic stress categories contained multiple genes encoding cold shock proteins, dehydrin, and G-protein, which may be induced by various stresses during development. Furthermore, a large proportion of elongation factors (EF) such as *EF1-alpha*, *EF1-beta*, *EF1-delta*, *EF1-gamma*, *EF2*, elongation factor G, and elongation factor-Tu were contained in this cluster.These clusters comprised several genes encoding lipases, containing the GDSL motif and lipid-transfer protein, which is involved in the lipid metabolic process. Many genes in the cell organization category were related to the cytoskeleton, including several tubulin alpha-1/−2, tubulin beta-1, and kinesin genes. In addition, genes encoding cell division cycle protein 48, cell division control protein 6, and cell division control protein 45 were identified in this cluster. Furthermore, a number of genes associated with cellulose synthase, cell wall, and cell cycle were expressed in this phase, possibly indicating a major shift in the regulation of cell morphogenesis.

Subcluster_5_3 and subcluster_5_4 showed low expression peaks in phase V_DON, followed by rapid increases from phase VG_DON to phase BH_DON. To evaluate the transcriptional changes associated with the morphologic changes after vernalization, we examined a number of genes in these clusters via enrichment in GO and KEGG. Genes in these clusters were predominantly enriched in phenylpropanoid and starch and sucrose metabolism in KEGG; also, the pathways of plant hormone signal transduction were involved. The functional category protein binding, transferase activity, and catalytic activity were revealed via GO enrichment. An overrepresentation of transcription factors were enriched, including AUXIN(AUX)/INDOLE-3-ACETIC ACID (*IAA*) family genes *IAA6*, *IAA13*, *IAA21*, and *IAA31*, the AUXIN RESPONSE FACTOR1(*ARF1*), and WRKY transcription factor family genes *WRKY 27*, *WRKY 30*, *WRKY 40*, *WRKY 46*, and *WRKY 50*. Otherwise, the primordia in embryos and flower development related transcription factors, no apical meristem (NAM) also involved in this cluster.

Subcluster_5_5 and subcluster_5_6 showed unique expression patterns, which have expression peaks in phases V_DON and VG_DON by negative regulation, and were also strongly expressed in phase BH_DON via positive regulation. These clusters contained a large number of genes involved in the protein process in the endoplasmic reticulum and phenylpropanoid biosynthesis, which played a role in cell morphogenesis and structural molecule activities. Subcluster_5_5 contained an overrepresentation of transcription factor induced by various abiotic factors, including ethylene-responsive transcription factor11 (*ERF11*), ethylene-responsive transcription factor34 (*ERF34*), and WAX INDUCER1 (*WIN1*).

### Coexpression network analysis

**Fig. 4:**
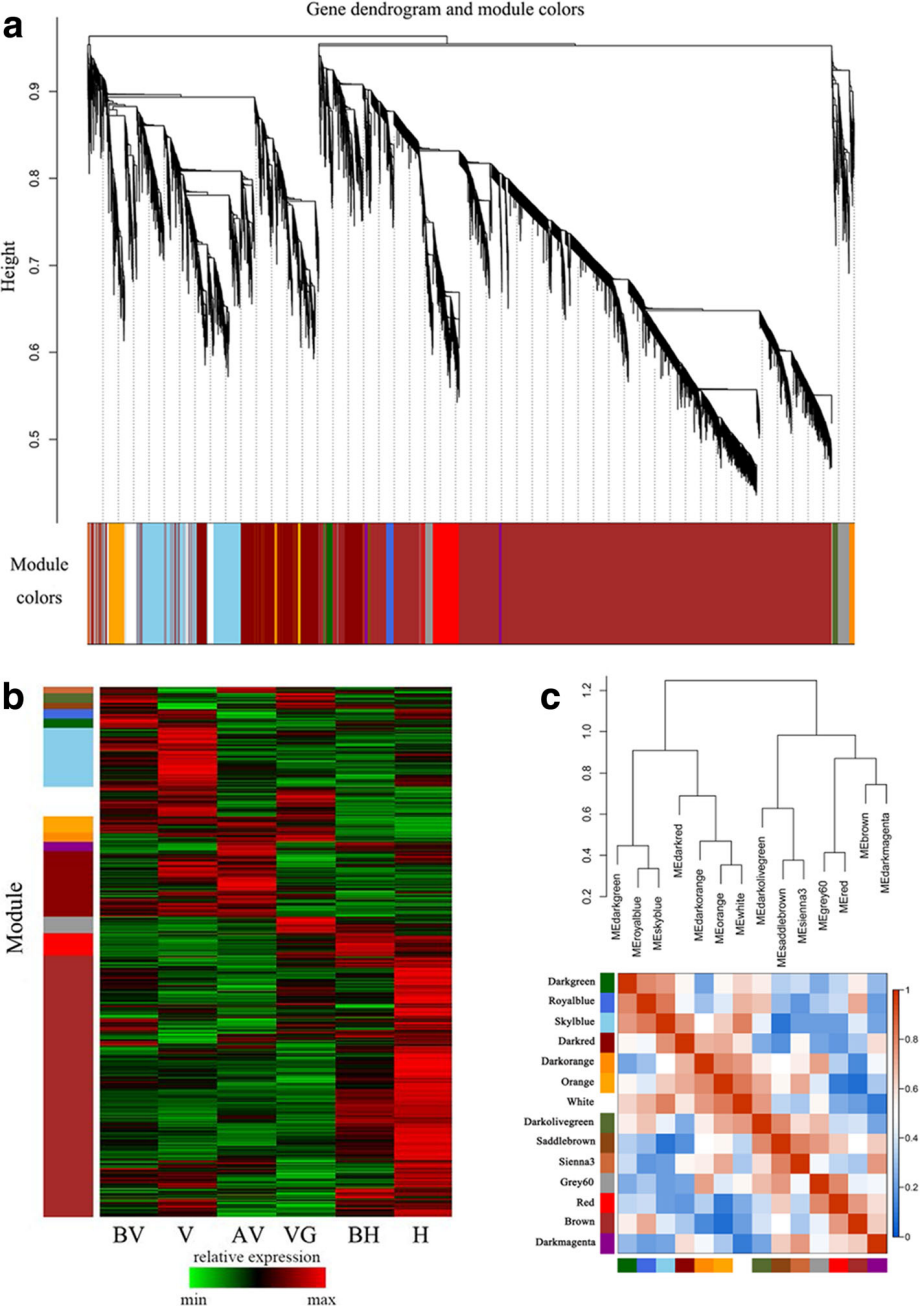
WGCNA of genes at six stages. **a** Hierarchical cluster tree shows coexpression modules, identified via WGCNA. Each leaf in the tree represents one gene. Major tree branches constitute 14 modules labeled by different colors. **b** This heatmap shows the gene relative expression of different modules in six stages, the y-axis represents the relative expression of modules and the x-axis shows the different stages. **c** Visualization of the eigengene network represents the relationships among the modules and the clinical trait weight. The hierarchical clustering dendrogram of the eigengenes shows the relationships among the modules. Heatmap shows the correlation of different modules, and the deeper red color represents the higher correlation. Sample labels are as follows: BV, before vernalization; V, vernalization; AV, after vernalization; VG, vegetative growth; BH, before heading; H, heading

To further investigate the coexpression network of candidate genes, a weighted correlation network analysis (WGCNA) was adopted. Based on pairwise correlations and gene expression trends in all samples, coexpression networks were constructed using the normalized microarray expression data of these 25,071 probes from all 18 samples (three biological replicates for six time points) via R library. Different color represents a specific module, containing a cluster of highly correlated genes. This analysis resulted in 14 distinct modules (Fig. 4a). Remarkably, 6 out of 14 coexpression modules comprise genes that are highly expressed in a single stage (Fig. 4b). For example, the brown module, included 14,121 genes that were significantly enriched in stage H_DON. Alignment of these genes in GO classes covered terms associated with signal transduction, transcription regulation, carbohydrate metabolic, and photosynthesis. Specific terms relevant to floral bud development and heading were abundant, such as cell wall synthesis and remodeling, including cell wall pectin biosynthetic processes, cell wall macromolecule catabolic processes, and cell wall modification. Moreover, the terms of cell morphogenesis, cell proliferation, cell growth, and cell division were also abundant in this module. These terms were identified and can be further used to predict key regulatory genes involved in floral bud development and heading. The red module contained the genes mainly expressed during stage BH_DON and GO terms in this module were similar to the brown module, which may demonstrate that these two modules shared a common gene network and thus respond to similar physiological processes. In the same way, we can identify the genes that significantly impact vernalization inducted by low temperatures. The genes in saddlebrown module and sienna 3 module, expressed lower than other modules, however, the skyblue module contained genes with higher expression in stage V_DON and may provide information for the key regulators in vernalization.

In addition, the correlation in different modules was also considered, and 7 broad clades were identified in 14 modules (Fig. 4c). A heat map showed that there was a high degree of correlation among the darkgreen module, the royalblue module, and the skyblue module. The genes in these modules were massively expressed in stage BV_DON and V_DON. GO analysis in these modules identified series terms, involved in response to stress, signal transduction, and protein phosphorylation, indicating that these clusters may participate in the process of response stimulation and may guide vernalization. Some genes were coexpressed in the grey60 module, darkorange module, and orange module. In addition, the heatmap showed that the brown, red module, and darkmagenta modules coexpressed in stages BH_DON and H_DON. A host of transcription factor families were identified in these modules, such as the zinc finger transcription factor family (MIZ-type, C2H2-type, and C3HC4-type), the WRKY transcription factor family, and the NRT1/PTR family. In particular, we identified a series of genes that encode the YABBY protein, which plays a role in reproductive development [33].

### The expression pattern of transcription factors and regulatory motifs at different stages

**Fig. 5:**
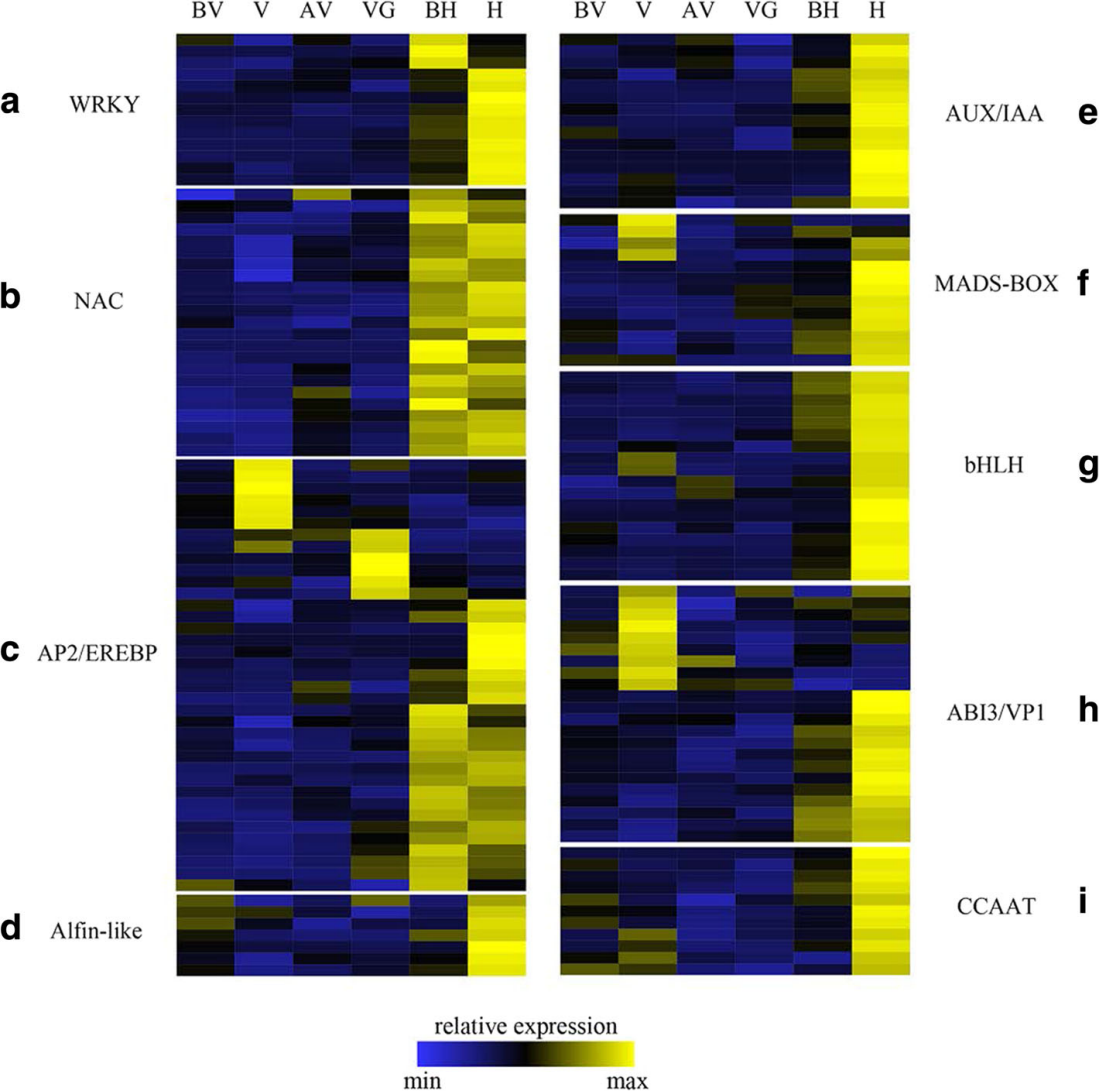
Transcription factor profiling according to RNA-seq data. The heat map shows the expression of transcription factor families that are overrepresented among coexpression clusters. The y-axis represents transcription factor families: WRKY transcription factor families (**a**); NAC transcription factor families (**b**); AP2/EREBP transcription factor families (**c**); Alfin-like transcription factor families (**d**); AUX/IAA transcription factor families (**e**); MADS-BOX transcription factor families (**f**); bHLH transcription factor families (**g**); ABI3/VP1 transcription factor families (**h**); CCAAT transcription factor families (**i**), and the x-axis shows the different stages. Sample labels are as follows: BV, before vernalization; V, vernalization; AV, after vernalization; VG, vegetative growth; BH, before heading; H, heading

A total of 3079 transcription factors were identified and classified in 74 transcription factor families. We are concerned about the following nine families: WRKY, NAC, AP2/EREBP, Alfin-like, AUX/IAA, MADS-BOX, bHLH, ABI3/VP1 and CCAAT. In our data, we noticed that some transcription factor families expressed in specific period. For example, the Alfin-like transcription factor family peaked in phase H_DON and were only minor expressed in other phases (Fig. 5d). The basic Helix-Loop-Helix (*bHLH*) family, comprising transcription factors that are known to be active during flower development in *A. thaliana,* peaked in the phase of H_DON [34] (Fig. 5g). The nuclear transcription factor Y contains the three subunits *NF-YA*, *NF-YB*, and *NF-YC* that bind with highly specific CCAAT motifs in a variety of genes. We found these transcription factors overrepresented in phase H_DON (Fig. 5i). In addition to the above three transcription factor families, the AUX(AUXIN)/IAA(INDOLE-3-ACETIC ACID) transcription factors also have low expression level in other phases and with high expression level in phase H_DON (Fig. 5e).

Otherwise, some transcription factor families have two expression peak among developmental stages. ABI3/VP1 transcription factor family encoded the B3 domain-containing protein, were the key components of auxin signaling include the AUX/IAA coreceptors and ARF transcription factors. Our data showed this type of transcription factors was strongly expressed in phase V_DON and phase H_DON (Fig. 5h), as well as the MADS-box transcription factors (Fig. 5f). The WRKY transcription factor family was widely distributed in the regulation of the transcriptional response to stress. In our data, we found the WRKY transcription factors expressed during different phases, especially in phase BH_DON and H_DON (Fig. 5a), which have the similar expression pattern of NAC transcription factor family (Fig. 5b). Unlike other transcription factor families, AP2/EREBP had expression peaks in phase V_DON (cold stress), AV_DON, and VG_DON (heat stress), as well as in BH_DON and H_DON (floral development) (Fig. 5c).

### Identification of expression patterns of key flowering regulators in Orchardgrass

**Fig. 6:**
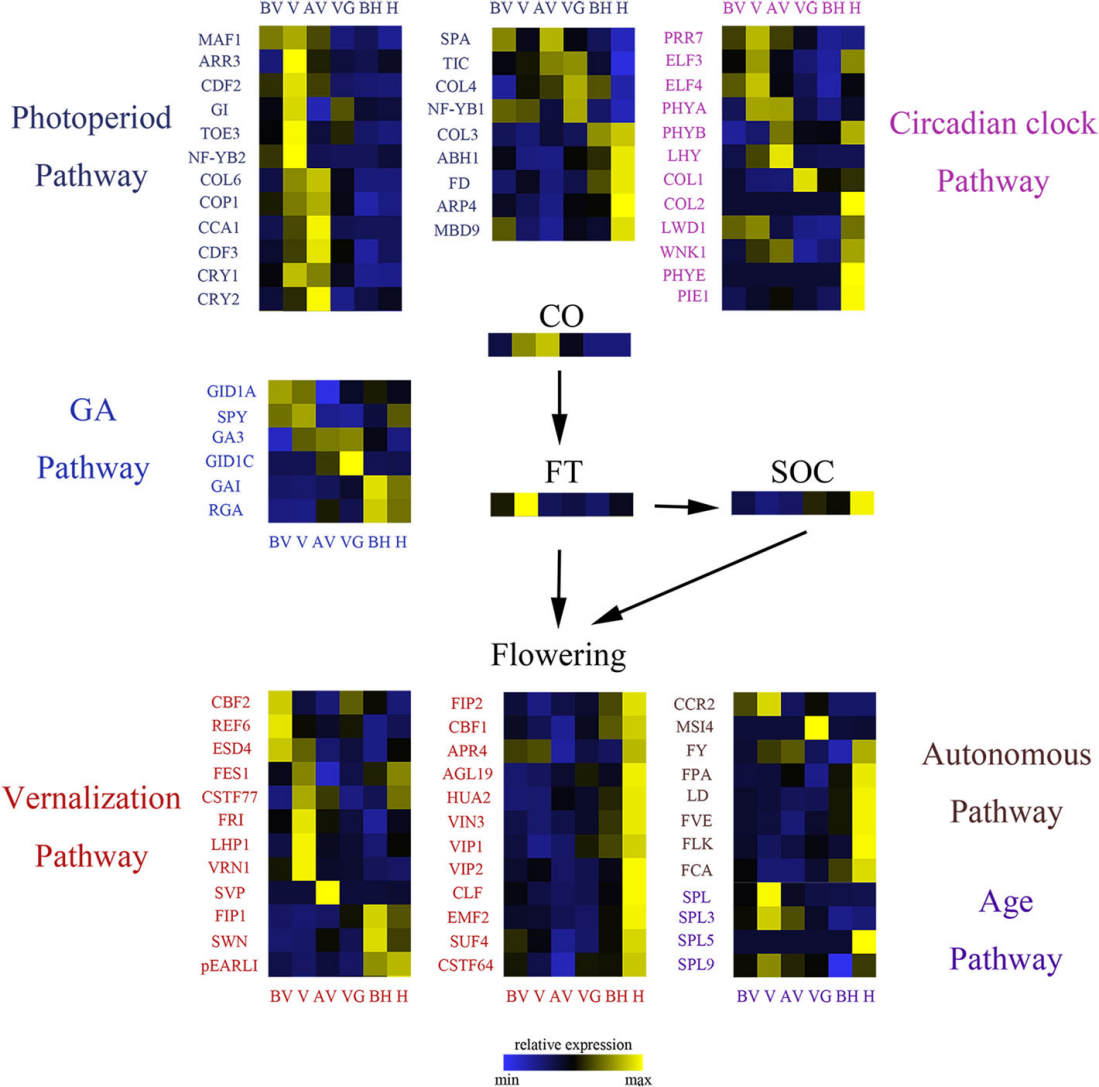
Putative schematic network of bolting and flowering regulation in orchardgrass. Arrows indicate positive regulation. The heat map shows the relative expression of candidate genes in different stages, the y-axis represents identified candidate genes, and the x-axis shows the different stages. These identified candidate genes are involved in various flowering pathways according to previous reports. Sample labels are as follows: BV, before vernalization; V, vernalization; AV, after vernalization; VG, vegetative growth; BH, before heading; H, heading

Due to the importance of flowering for plant reproduction, many studies report the gene function and genetic network of flowering regulation in *A. thaliana* [35]. And more than 200 flowering associated genes have been identified and characterized [36, 37]. In this study, we captured these candidate genes in multiple different flowering-related pathways to understand the dynamic change during the developmental switch. A total of 77 reported flowering-related genes were identified in our transcriptome data and the great majority of these were located in the pathways of vernalization and photoperiod (Fig. 6, Additional file 11: Table S3, Additional file 12). According to previous research, 24 potential genes including *VRN1*, *VIN3*, *FRI*, *SVP*, *VIP1*, *VIP2*, and *SUF4* were implicated in vernalization. Furthermore, 22 candidate genes were involved in the photoperiod pathway, including *MAF1*, *CDF2*, *NF-YB1*, *NFYB2*, *TIC*, *COL*, and *FD*. In addition, the pathways of circadian clock, autonomous, GA, and aging contained 10, 8, 6, and 4 candidate genes, respectively. Most vernalization pathway related genes with high expression levels during phase 2 and phase 5 include *VRN1* and *FRI*. *VRN1* is a key regulator in the vernalization pathway. The high expression level of *VRN1* in phase 2 matches the observation that low temperatures induce *VRN1* [38]. In *A.thaliana*, *FRI* is an upstream regulator, repressing the expression of integrators *FT* via activating *FLC*, thus causing late flowering. Our data shows the *FRI* with high expression during phase 2, followed by a drastic decrease during phase 3. This result is consistent with research, showing that cold stress induces the degradation of *FRI* [15]. Within the photoperiod pathway, genes are enriched in short-day conditions (phase 2) and long-day conditions (phase 5), suggesting that genes in this pathway may respond to different length of hours of sunlight.

**Fig. 7:**
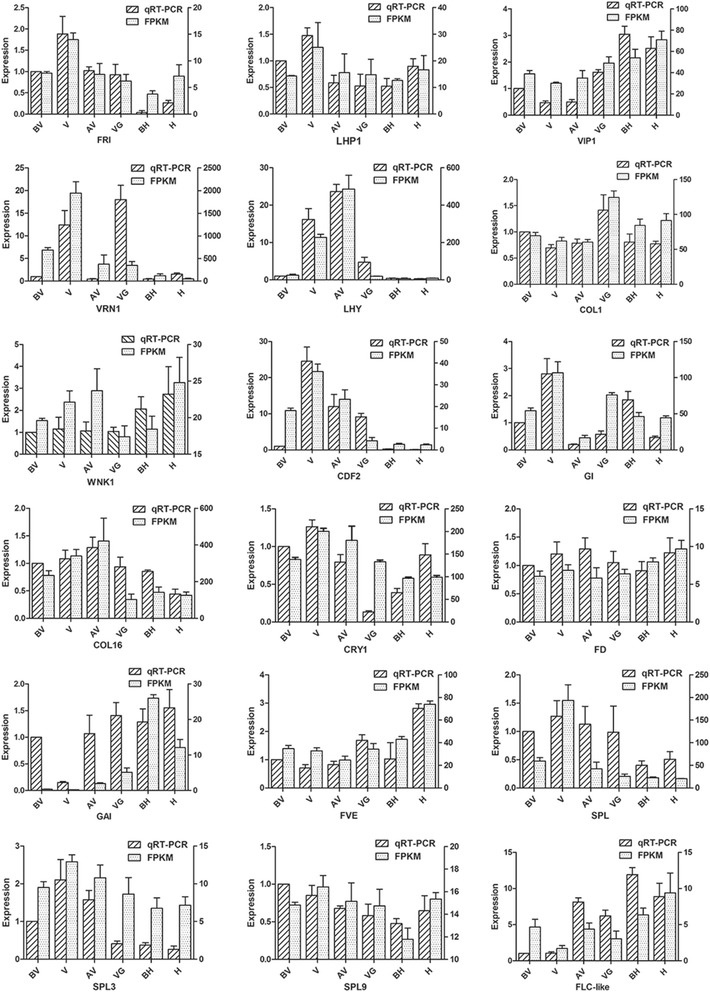
qRT-PCR validation. The abscissa represents the different sampling time, ordinate represents the expression. The bar with oblique stripes represents the relative expression base on qRT-PCR results and the bar with faillette base on RNA-seq results. The *bottom* title represents the different genes. Sample labels are as follows: BV, before vernalization; V, vernalization; AV, after vernalization; VG, vegetative growth; BH, before heading; H, heading

To confirm the RNA-seq results, 18 unigenes were selected for qRT-PCR. In general, the relative expression based on qRT-PCR was consistent with the sequencing data (Fig. 7). But there were also some inconsistent trends between qRT-PCR and sequencing data at specific stages. For example, the qRT-PCR results showed that the expression of *COL1* and *SPL3* were down-regulated in stage BH_DON comparing to stage H_DON, while the sequencing data showed up-regulated (Fig. 7F and P). In addition, the expression of *CRY1* was up regulated from stage VG_DON to BH_DON base on the qRT-PCR results, while the sequencing data showed the down regulation (Fig. 7K). On the contrary, the expression of *GAI* was down-regulated in stage VG_DON relative to BH_DON, while the sequencing data showed up regulation(Fig. 7M).

## Discussion

Flowering time is one of the major factors affecting forage quality in orchardgrass. A comprehensive transcriptome profile of the critical periods, comprising vernalization, young spike differentiation, and flower development was obtained via RNA-seq. DEGs were identified and functional characteristics also elaborated. The analysis of expression patterns during six key developmental stages revealing the abundant diversity in gene expression. Stage-specific profiles indicated a dramatic shift in gene expression during different developmental stages, and while many genes were expressed constitutively, very few of these were expressed evenly throughout all stages. The analysis captured the distinct molecular characteristics during vernalization and flower formation in orchardgrass, which could be further explored to help understand flowering regulation in response to seasonal cues in grass.

### Genome-wide expression profiling uncovered developmental modules in vernalization and flower formation

The number of DEGs between the adjacent sampling points indicates that there were much more DEGs in phase 4 (P4) than any other phases, and more remarkably, phase 5 only had the least DEGs. This demonstrated that P4 involves far molecular processes and most of these processes have had a regulatory effect before heading. This result agrees with the markedly morphological alterations during P4 (inflorescence formation) and P5 (heading). The plant perception of vernalization and outgrowth of floral buds, involved numerous processes, both temporal and spatial. GO and KEGG analysis of DEGs throughout all five phases showed that the processes focused differently, which also supported this notion. The protein related processes, such as protein phosphorylation and kinases, and protein modification, were highlighted during P1, while the functional hormone related categories were also overrepresented. Protein phosphorylation plays a central role in regulating many cellular processes in eukaryotes. In particular, protein phosphorylation is a major currency of signal transduction pathways [39]. This result indicates that the plant responded to multiple environmental stimuli during this phase. Compared to P1, the functional categories of carbohydrate metabolic processes and sucrose metabolic processes were significantly enriched and previous research suggests that these two processes may associate with gene expression throughout dormancy in crown buds [40]. Cold temperature was involved in breaking dormancy, inhibiting shoot growth, and inducing flowering competence [41]. These are critical phases of plant floral bud formation and development during P4 and P5. The functional categories including cell wall, cytoskeletal, polysacharide metabolic, and catalytic activity, collectively coordinate organogenesis. These biomolecular processes potentially relate to subtle morphological changes. KEGG analysis of DEGs highlighted the otherness of temporal and spatial.

We found various pathways that focus differently throughout developmental phases. In P1 and P2, plant hormone signal transduction, NOD-like receptor signaling, and MAPK signaling were emphasized. Plant hormones exhibit strong functions in response to endogenous signals and environmental cues. Furthermore, the ubiquitin biosynthesis pathways were also identified in this phase, and ubiquitin-mediated proteolysis has been shown to regulate the different steps of plant hormone signal transduction [42]. Ethylene is an important plant stress and defense hormone and MAPK modules act downstream of the receptors, thus mediating ethylene signaling [43]. Recently, researchers suggested that the starch and sucrose metabolism, carbon metabolism, and plant hormone signal transduction all mediate the timing of transition from vegetative to reproductive phase [44]. This report is consistent with our KEGG enrichment results during P3 and P4. The functional category of circadian rhythm-plant, flavonoid biosynthesis, and starch and sucrose metabolism were enriched in most phases, suggesting that biological processes during specific stages and non-specific stages play equally important roles.

### Stage-specific analysis outlines the core molecular processes related to vernalization and flower formation

Coexpressed gene cluster analysis illustrated the temporal distribution of core molecular events during vernalization and floral bud formation. Our results suggested that transcriptome specialization is established at a specific stage for several biological processes. At vernalization stage, genes related to signal transduction and stress response were overrepresented. When temperature dropped, several mechanisms have been described to enhance the freeze tolerance, including changes in lipid composition, increases in active-oxygen-scavenging enzymes, anthocyanin accumulation, and carbohydrate metabolism, which are consistent with our transcriptome sequencing results [45]. Signal transduction includes protein phosphorylation, calcium ion signaling, plant hormone signaling pathways, and many metabolic processes. These various pathways reflect the biological processes of plants at this stage to receive and cope with the external multiple stimuli. For perennial grasses, vernalization induced by cold stimulation is critical for plant flowering. We can capture potential genes that mediate vernalization in these clusters. Our results also indicated that photosynthesis-related genes are show strong stage dependency and with high expression levels during V_DON stage, which may indicate these kind of genes are tightly co-expressed. Under normal conditions, low temperature causes photoinhibition of photosynthesis [46]; however, the elevated expression of these genes is consistent with previous studies. This suggests that the photosynthetic contribution (reproductive assimilation) of reproductive structures propagates production and influences flower initiation [47]. Previous research demonstrated that CO_2_ assimilation may play a regulatory role during early reproductive stages [48].

To identify genes associated with flower formation, we examined genes with expression peak in BH_DON and H_DON. Several clusters highlighted a number of specialized features of transcriptome data. Subclusters_1_6, subcluster_5_1, and subcluster_5_3 revealed the importance of cell wall remodeling by the active cellulose metabolism in the BH_DON stage, which agrees with reports of cellulose as a cell wall polymer as found in eukaryotes [49]. Plant cell wall-loosening proteins promote cell growth and are essential for floral bud initiation and differentiation. During the plant cell expansion process, microfibers are usually functional in the direction of cell expansion, which were laying down secondary cell walls [50]. Furthermore, genes related to cellular aromatic compound metabolic processes, cell wall modification, cell wall macromolecule catabolic processes, and cell wall biogenesis also play pivotal roles in cell growth [51]. In addition, genes within cell morphogenesis, cell division, cell cycle, and cell differentiation categories with higher expression levels, are indicative of multiple processes that actively coordinate tissue development and organ morphogenesis during floral bud formation [52].

This is exemplified by subclusters_5_5 and subcluster_5_6, which indicate a functional partition of different developmental stages by the expression pattern. These subclusters had higher expression during stages V_DON and VG_DON compared to other stages. The subclusters were characterized by genes related to signal transduction and stress responses, likely reflecting the fact that the external environment significantly influenced plant growth during these stages. A distinct gene set associated with cell proliferation and differentiation during BH_DON and H_DON provides evidence that genes with specific function express during a specific period. In other cases, expression preference exists even though gene clusters were expressed throughout the growth stage.

WGCNA analysis enabled the identification of specific modules during critical developmental stages. The skyblue module and darkmagenta module connect with vernalization induction in stages V_DON and AV_DON. Furthermore, the grey60 module, red module, and brown module, may contain numerous genes that are associated with growth transduction, floral organ development, and flowering control, respectively. Furthermore, WGCNA also provided evidence in the interaction of different modules during certain stages. This clearly revealed a network of genes instead of individual genes, which may help to uncover the molecular mechanisms underlying vernalization and flowering. Furthermore, the conjoint analysis of WGCNA and clustering analysis supplied a more efficient method to search core regulators in diverse and abundant data.

### Transcriptional regulation among different stages

An analysis of genes that are associated with transcription regulation showed that TFs play an important role during vernalization and floral bud formation. Expression profiles during different developmental stages suggest temporal specialization of TFs and the data set can be used to identify key TFs relevant for flowering time control. For example, WRKY, MADS-BOX, and ABI3/VP1 transcription factor families were found to preferentially accumulate during vernalization, floral organ formation, and heading, which could be part of the transcriptional regulatory complex regulating stress response and flower development. Several WRKY transcription factors expressed in phase BH_DON and H_DON, which is consistent with their reported role in *Miscanthus*, including *WRKY 12*, which participates in cell wall formation and promotes flowering [53]. *WRKY20*, *WRKY23*, *WRKY30*, *WRKY40*, *WRKY50*, *WRKY53*, *WRKY57*, and *WRKY70* were involved in ABA and jasmonic acid signaling pathways in moderating flowering [54]. The ABI3/VP1 transcription factor family encoded the B3 domain-containing protein and play a central role in embryogenesis and dormancy by regulating the gene expression during the plant embryo maturation via ABA responsive elements or conserved RY repeats [55]. In a previous study, the B3 domain-containing protein demonstrated to participate in the early development of flowers [56], including the reproductive meristem gene (REM) [57], which was preferentially expressed in reproductive meristems.

The MADS-box family has been defined on the basis of primary sequence similarity amongst numerous proteins [58]. In a previous study, the MADS-box was considered to play an important role during the origin and evolution of flower development [59]. MADS-box transcription factor suppressor of overexpression of CO1 (*SOC1*) signals from the GA-dependent pathway, which influences the flowering time during short days [60]. The presence of MAD-box transcription factors is consistent with the overrepresentation of the regulation of transcription functional categories.

In addition, Alfine-like and CCAAT transcription factor family were overrepresented in stages BH_DON and H_DON. Alfin-like transcription factors encoded several PHD finger proteins, including vernalization insensitive 3 (*VIN3*) and vernalization 5 (*VRN5*) [61, 62], which mediated both vernalization and photoperiod. The nuclear transcription factor Y contains the three subunits *NF-YA*, *NF-YB*, and *NF-YC* that bind with highly specific CCAAT motifs in a variety of genes. The *NF-YA* regulate transcriptionally and post transcriptionally to promote drought resistance [63, 64]. *NF-YB* plays a role in the regulation of the flowering time in *A. thaliana* [65]. Furthermore, members of *NF-YC* can physically interact with constans (*CO*), and are genetically required for CO-mediated floral promotion.

Previous research demonstrated basic helix-loop-helix (bHLH) transcription factor family to participate in controlling cell proliferation and development of specific cell lineages [66]. Our study found bHLH TFs significant enriched during booting stage and heading time, suggesting this type of transcription factor family may play a similar role in the regulation of flower formation and heading. NAC transcription factors (*NAM*, *ATAF*, and *CUC2*) were described during recently years [67], only a proportion of NAC proteins have been studied. NAC genes are tightly associated with embryonic, floral, and vegetative development [68]. We found NAC genes concentrated expression in phase BH_DON and H_DON in our data, meaning that this type of TFs are produced to join the pathways that lead to flower organ formation and flowering, which was consistent with the previous reports.

The ethylen-responsive transcription factor (*ERF*) is an important member of *AP2/EREBP* and encoded a type of AP2 containing protein. Many ERFs have been reported to be involved in the regulation of floral development and stress response [69]. This transcription factor family consists of a large quantity of genes that encode IAA, which have an important function in floral organ development and plant growth regulation [70]. Hormone-related TF families *AUX/IAA* and *AP2/EREBP* were found in abundance at stage V_DON, VG_DON, BH_DON and H_DON, this suggested that ethylene biosynthesis and signaling have great function in stress response and are particularly associated with flower development [71]. The auxin response factor (*ARF*) family comprises transcription factors that are known to act during different phases in floral organ formation and are operated by a complex transcriptional network [72]. Furthermore, ARF transcription factors contribute to the response of abiotic stresses, containing *ARF7* and *ARF19*. *ARF* is an important regulator of auxin activity, and these genes have maximal expression during stage H_DON, suggesting common centers for auxin biosynthesis and transduction during heading time. The diverse expression profiles of gene related hormone signaling may constitute the basis for this stage-specific response, also supplying evidence for the importance of hormones in vernalization and flower development. Timing of CAB expression 1(*TOC1*) is a widespread study circadian clock gene belongs to AP2/EREBP family, which has been suggested to be a component of the central oscillator of controlling flowering time in *A. thaliana*. *TOC1* is designated as *Arabidopsis* pseudo-response regulators (*APRR1/TOC1),* a circadian-associated transcription factor family. Other than *APRR1/TOC1*, most APRR family members have been implicated in the mechanisms underlying the circadian rhythm; in particular *APRR5*, *APRR7*, *ARR4*, *ARR3*, and *ARR9* in *A. thaliana* [73], *OsPRR73* and *OsPRR95* in rice [74], and *SbPRR37* in sorghum [75].

Most MYB proteins function in a variety of plant-specific processes especial in controlling plant development, responses to stresses [76]. The MYB-related gene Circadian 1 (*CIR1*) was identified in the transcriptome data and has been reported to affect several circadian-regulated processes [77]. In addition, MYB proteins mainly participated in signal transduction such as phytochrome A signaling, jasmonate signaling, ABA signaling, and GA signaling, which is involved in a variety of processes [78, 79]. Otherwise, the photoperiod and the circadian clock pathway gene *STO* (*BBX24*) that belongs to B-Box Family can affect the key flowering time genes *FLC* and FT/SOC1, thus regulating the flowering time [80]. The zinc finger proteins constans-like (*COL6)* and the SBP-box gene family squamosa promoter binding protein-like3 (*SPL3*) [81], which are involved in flowering regulation were also identified in transcription factors examination.

### Key flowering regulators in orchardgrass

Studies on the flowering network of *A. thaliana* and other species uncovered several critical regulators for the integration of multiple flowering pathways, including *FT* and *SOC* [82]. These regulators are mediated by two dominant upstream genes, which implicate *FLC* in pathways of vernalization, autonomous, and aging. The previous study indicated the upstream gene *FLC* have negative regulation on *FT*. We found the *FLC* have low expression in vernalization stage and then increase gradually until heading stage. On the contrary, the FT have high expression in vernalization.while lowly expressed in other stages.This results were consistent with the existing conclusion that low temperature inhibit the expression of *FLC* and release the suppression for *FT*, which cause flowering transition [83]. Otherwise, the other upstream gene *CO* positively regulate the FT [84]. Our results showed that the *CO* and *FT* highly expressed in vernalization stage, which may provide support that the *CO* promote the expression of *FT* in flowering regulation. Identified these key regulators in RNA-seq data of *D.glomerata* demonstrated that different species may share a highly conservative flowering genetic network and several homologous critical candidate genes likely have a similar function. Furthermore, we present these flowering pathway related genes via expression dynamics, providing an intuitive display of when the genes are active during developmental phases. Furthermore, our results provide more essential information for functional analysis of flowering regulatory pathways in perennial grasses.

## Conclusions

In conclusion, an RNA-seq approach was utilized to investigate the patterns of gene expression during six key flowering developmental stages involved in vernalization and flower development, revealing novel networks and key regulators. Stage-specific profiling provided biological information of molecular events. This evidence included the process of signal transduction, stimulation of the vernalization response, of hormone control, cell proliferation, and differentiation in floral organ formation. Furthermore, this study added insight into the vital function of transcriptional factors in plant growth as well as valuable information for plant biology in the area of flower development. The WGCNA approach revealed a tightly co-expressed gene clusters and highly ordered gene expression networks that control plant growth. Our work highlighted the effectiveness of the clustering analysis intersected with the WGCNA analysis tool in multi-sample and high-volume data analysis.

## Additional files

Additional file 1: Figure S1. The photo of orchardgrass in different stages. Including stage before vernalization (BV_DON); vernalization (V_DON); after vernalization (AV_DON); vegetative growth (VG_DON); before heading (BH_DON); heading (H_DON). (TIFF 8381 kb)

Additional file 2: The primers information for qRT-PCR. (XLSX 16 kb)

Additional file 3: Figure S2. The crown at the bottom of stem. A, showed the overall view. B, showed the anatomical structure of the plant. C, showed the magnified structure. The white arrow point the crown. (TIFF 7662 kb)

Additional file 4: Table S1. RNA-seq statistics. (DOCX 17 kb)

Additional file 5: Figure S3. Statistics of de novo assembly of transcriptome. A, Transcript length distribution. B, Unigene length distribution. (TIFF 8933 kb)

Additional file 6: Table S2. Statistics of annotation analysis of unigenes. (DOCX 16 kb)

Additional file 7: Figure S4. The box-plot describing the FPKM distribution of expressed transcripts after filtering in different samples. Sample labels are as follows: BV, before vernalization; V, vernalization; AV, after vernalization; VG, vegetative growth; BH, before heading; H, heading. DON refers to the orchardgrass cultivated varity DONATA (Registered No.398). (TIFF 9497 kb)

Additional file 8: Figure S5. GO functional classification of DEGs in five pairwise sampling stages. Including stage V_DON vs stage BV_DON(A), stage AV_DON vs stage V_DON(B), stage VG_DON vs stage AV_DON(C), stage BH_DON vs stage VG_DON(D) and stage H_DON vs stage BH_DON(E). (TIFF 10209 kb)

Additional file 9: Figure S6. KEGG functional classification of DEGs in five pairwise sampling stages. Including stage V_DON vs stage BV_DON(A), stage AV_DON vs stage V_DON(B), stage VG_DON vs stage AV_DON(C), stage BH_DON vs stage VG_DON(D) and stage H_DON vs stage BH_DON(E). (TIFF 9769 kb)

Additional file 10: Data S1 SOM-clustering results. (RAR 14661 kb)

Additional file 11: Table S3. Identified flowering-related gene in orchardgrass. (DOCX 19 kb)

Additional file 12: The annotation information for flowering-related genes identified in orchardgrass base on RNA-seq data. (XLSX 39 kb)

## Abbreviations

ABA: Abscisic acid
ABI3/VP1: Abscisic acid insensitive 3 or maize ortholog viviparous 1
AP2/EREBP: Apetala2 and ethylene-responsive element binding proteins
APRR1: *Arabidopsis* pseudo-response regulators
ARF: Auxin response factor
AUX/IAA: Auxin/indole-3-acetic acid
AV: After vernalization
BH: Before heading
bHLH: Basic helix-loop-helix
BV: Before vernalization
ChIP-seq: Chromatin immunoprecipitation sequencing
CIR1: Circadian 1
CO: Constans
COG: Cluster of orthologous group
CUL3A: CULLIN3A
DEGs: Different expressed genes
DON: The orchardgrass varity DONATA
EF: Elongation factors
ERF: Ethylene-responsive transcription factor
FDR: False discovery rate
FLC: Flowering locus C
FPKM: Fragments Per Kilobase of transcript sequence per Million base pairs sequenced
FT: Flowering locus T
GA: Gibberellin
GLPs: Germins and germin-like protein
GO: Gene Ontology
H: Heading
HSP: Heat shock protein
IAA: Indole-3-acetic acid
ICE1: Inducer of CBF expression 1
iTAK: Identification and classification of plant transcription factors and protein kinases database
JA: Jasmonic acid
KEGG: Kyoto Encyclopedia of Genes and Genomes
KOBAS: Kegg Orthology Based Annotation System
KOG: Eukaryotic orthologous groups
LAF1: Long after far-red light 1
MADS: MCM1, AGAMOUS, DEFICIENS and SRF
MYB: Myeloblastosis, a transcription factor family
NAC: NAM, ATAF1,2, CUC2
NAM: No apical meristem
NIL: Near-isogenic
Nr: Non-redundant protein sequence
OsPRR73: *Oryza sativa* pseudo-response regulators
Pfam: Protein family
psbN: Photosystem II reaction centre N protein
QTL: Quantitative trait locus
REM: Reproductive meristem gene
RNA-seq: Ribonucleic acid sequencing
SA: Salicylic acid
SOC1: Suppressor of overexpression of CO1
SPL3: SBP-box gene family squamosa promoter binding protein-like3
Swiss-prot: Annotated protein sequence database
TFs: Transcription factors
TOC1: Timing of CAB expression 1
V: Vernalization
VG: Vegetative growth
VIN3: Vernalization insensitive 3
VRN: Vernalization
VRN5: Vernalization 5
WAP1: APETALA1(AP1)-like MADS box gene in wheat
WGCNA: Weighted correlation network analysis
WIN1: Wax inducer 1
