# Supplementary figures and images for: Comprehensive transcriptome analysis reveals distinct regulatory programs during vernalization and floral bud development of orchardgrass (Dactylis glomerata L.)
Source: BMC Plant Biol. 2017 Nov 22;17:216. doi: 10.1186/s12870-017-1170-8 (PMC5700690; doi:10.1186/s12870-017-1170-8)

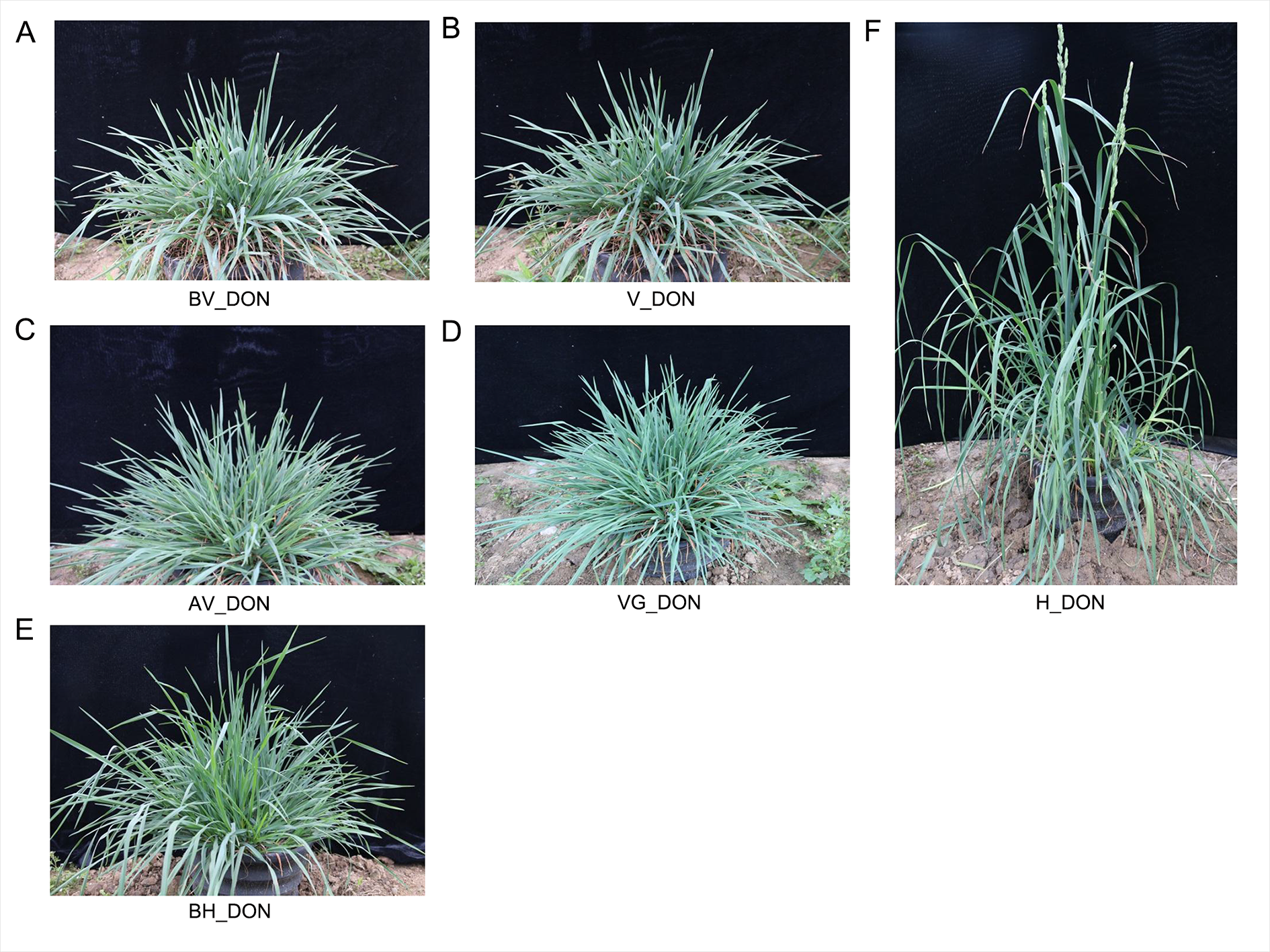

Supplement: Supplementary file 1 — The photo of orchardgrass in different stages. Including stage before vernalization (BV_DON); vernalization (V_DON); after vernalization (AV_DON); vegetative growth (VG_DON); before heading (BH_DON); heading (H_DON). (TIFF 8381 kb) [file 12870_2017_1170_MOESM1_ESM.tif]

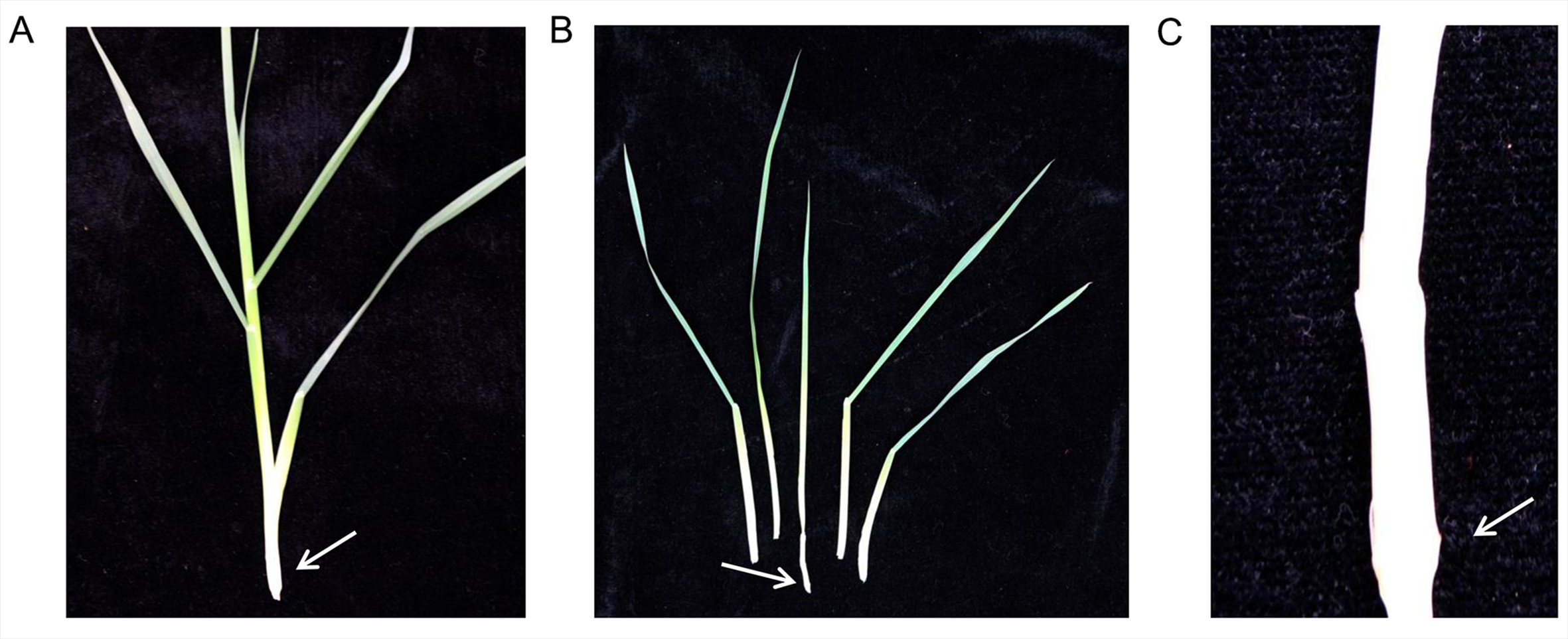

Supplement: Supplementary file 3 — The crown at the bottom of stem. A, showed the overall view. B, showed the anatomical structure of the plant. C, showed the magnified structure. The white arrow point the crown. (TIFF 7662 kb) [file 12870_2017_1170_MOESM3_ESM.tif]

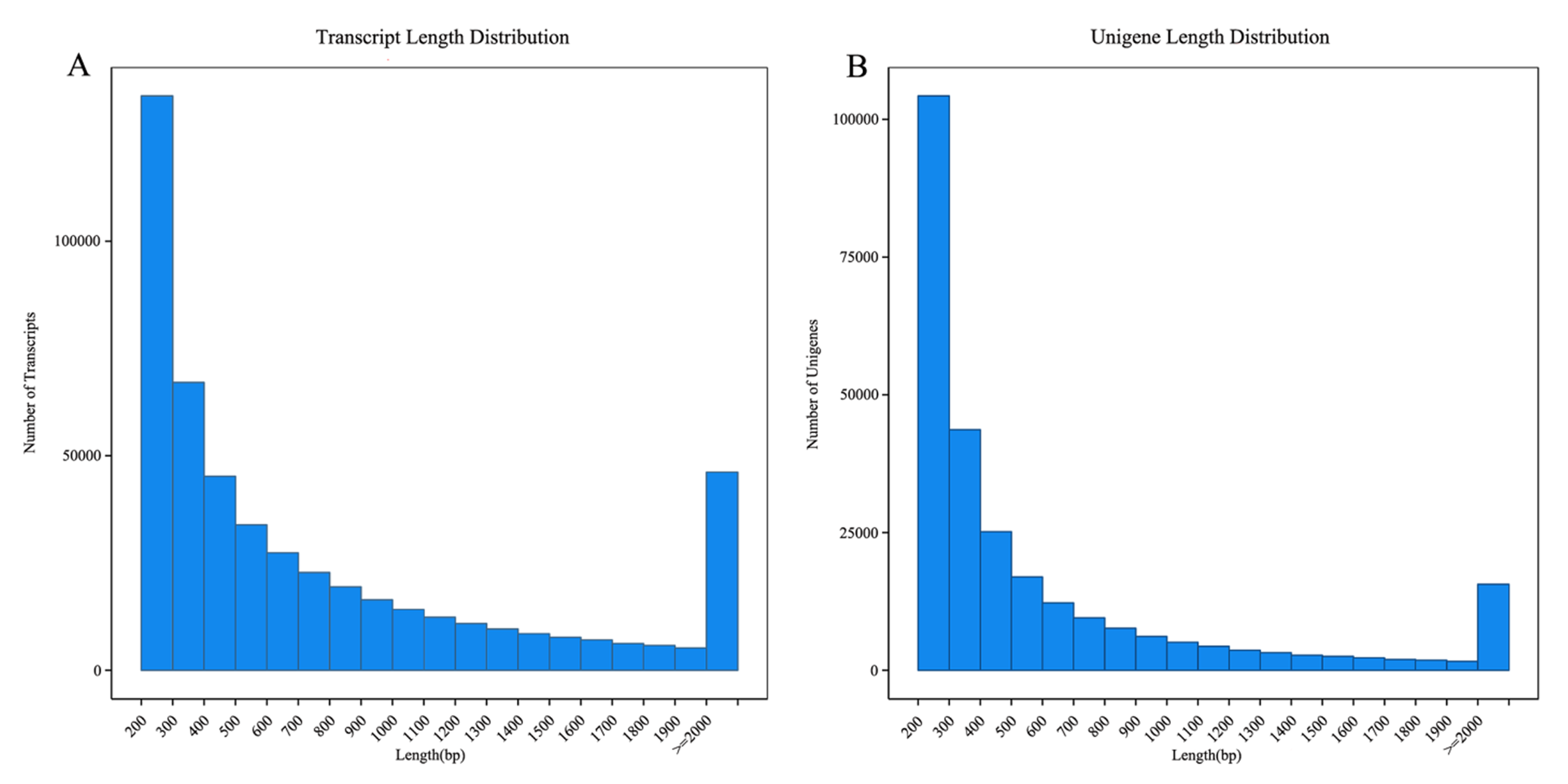

Supplement: Supplementary file 5 — Statistics of de novo assembly of transcriptome. A, Transcript length distribution. B, Unigene length distribution. (TIFF 8933 kb) [file 12870_2017_1170_MOESM5_ESM.tif]

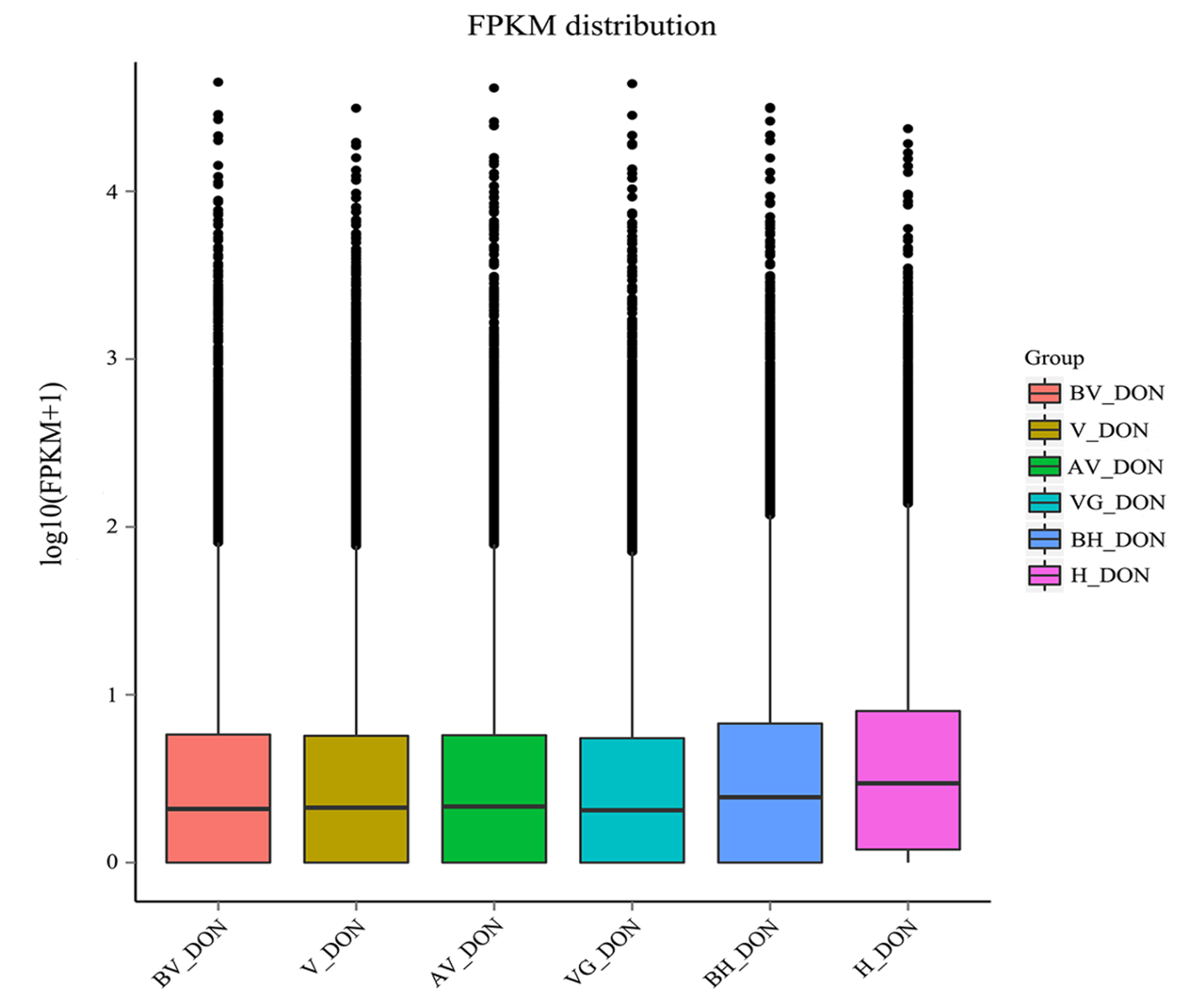

Supplement: Supplementary file 7 — The box-plot describing the FPKM distribution of expressed transcripts after filtering in different samples. Sample labels are as follows: BV, before vernalization; V, vernalization; AV, after vernalization; VG, vegetative growth; BH, before heading; H, heading. DON refers to the orchardgrass cultivated varity DONATA (Registered No.398). (TIFF 9497 kb) [file 12870_2017_1170_MOESM7_ESM.tif]

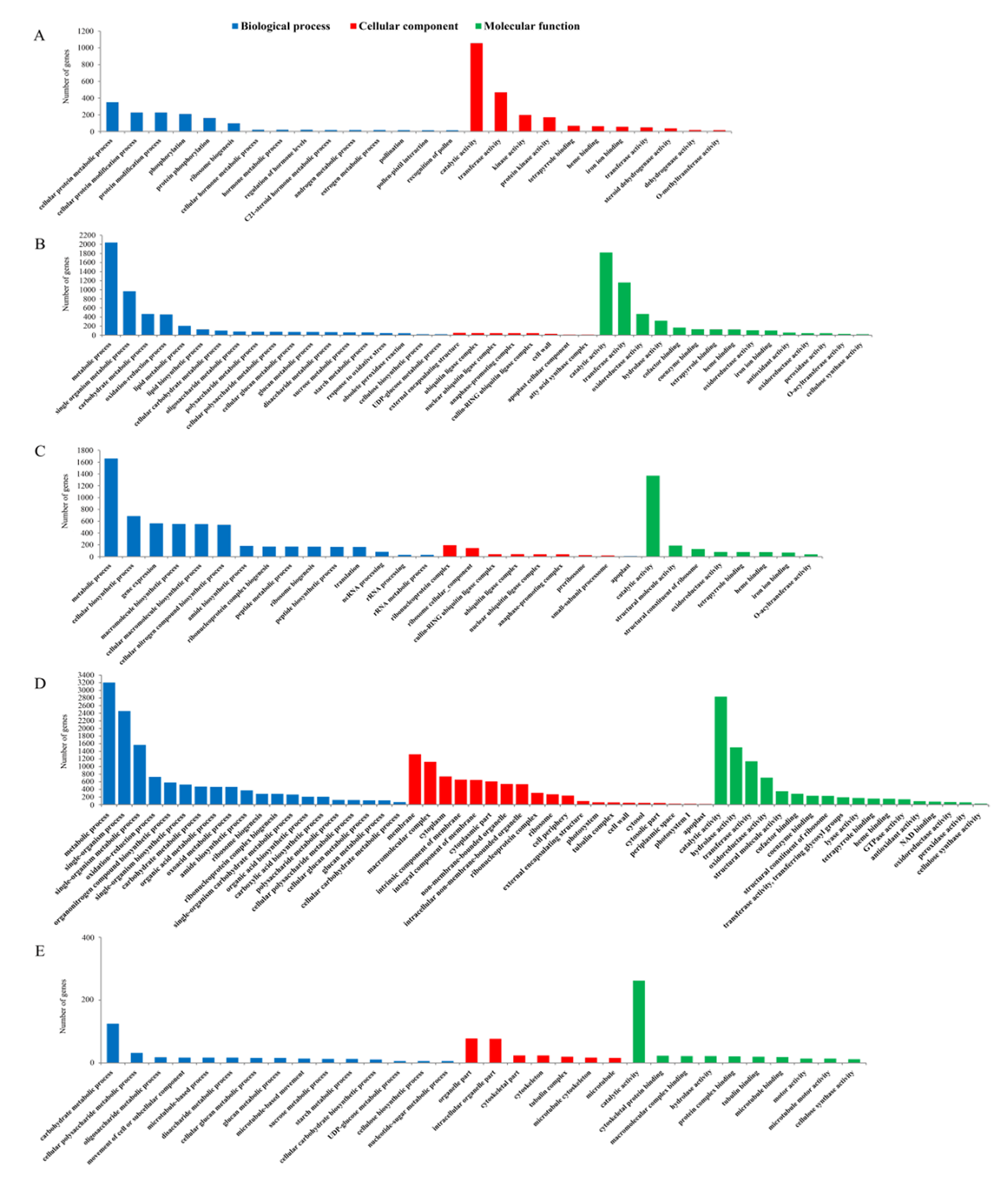

Supplement: Supplementary file 8 — GO functional classification of DEGs in five pairwise sampling stages. Including stage V_DON vs stage BV_DON(A), stage AV_DON vs stage V_DON(B), stage VG_DON vs stage AV_DON(C), stage BH_DON vs stage VG_DON(D) and stage H_DON vs stage BH_DON(E). (TIFF 10209 kb) [file 12870_2017_1170_MOESM8_ESM.tif]

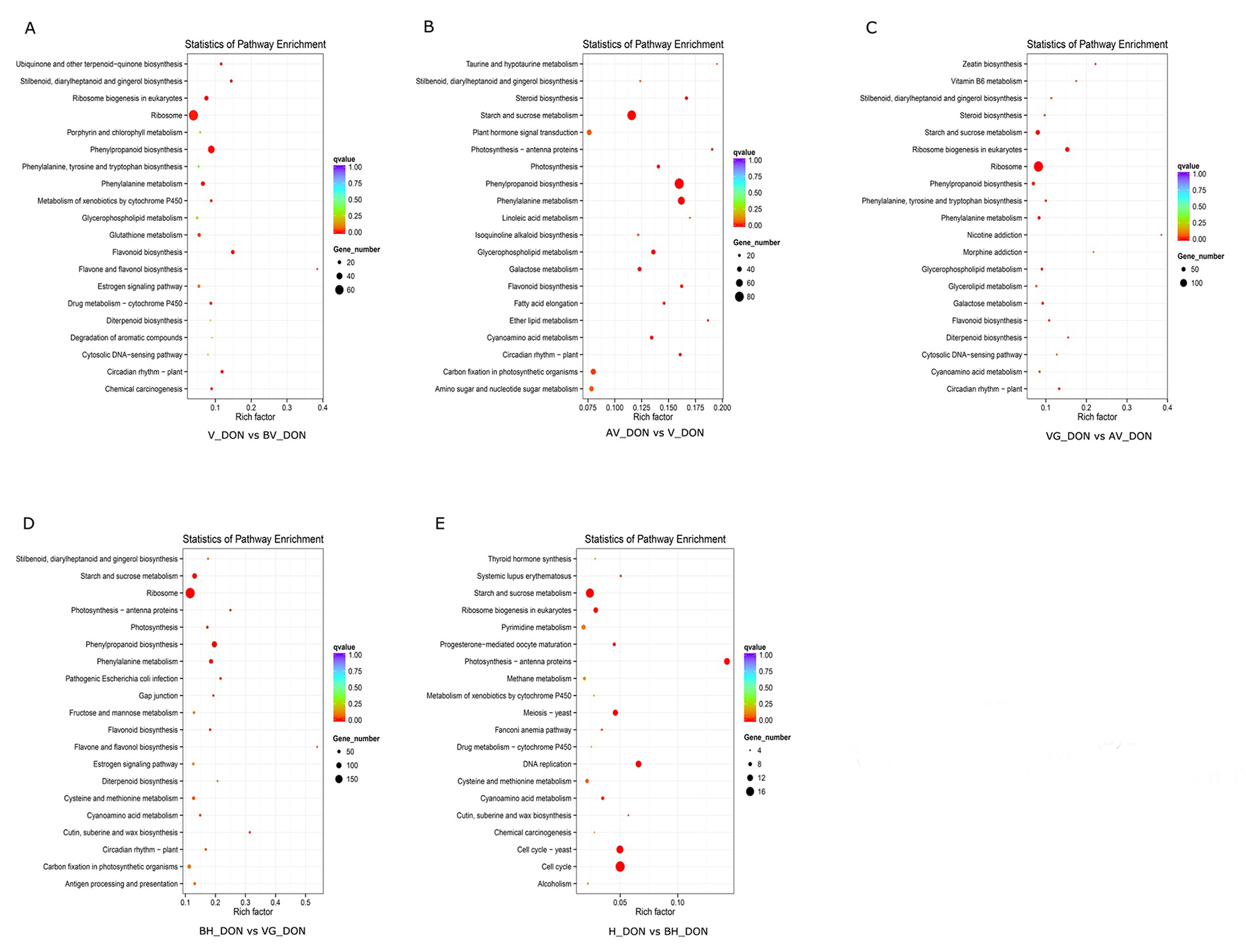

Supplement: Supplementary file 9 — KEGG functional classification of DEGs in five pairwise sampling stages. Including stage V_DON vs stage BV_DON(A), stage AV_DON vs stage V_DON(B), stage VG_DON vs stage AV_DON(C), stage BH_DON vs stage VG_DON(D) and stage H_DON vs stage BH_DON(E). (TIFF 9769 kb) [file 12870_2017_1170_MOESM9_ESM.tif]
