# Supplementary material for: Comprehensive transcriptome analysis reveals distinct regulatory programs during vernalization and floral bud development of orchardgrass (Dactylis glomerata L.)
Source: BMC Plant Biol. 2017 Nov 22;17:216. doi: 10.1186/s12870-017-1170-8 (PMC5700690; doi:10.1186/s12870-017-1170-8)
Supplement: Supplementary file 4 — RNA-seq statistics. (DOCX 17 kb) [file 12870_2017_1170_MOESM4_ESM.docx]

| Sample | Raw Reads | Clean reads | Total mapped | Q30(%) | GC(%) |
| --- | --- | --- | --- | --- | --- |
| BV_DON_1 | 62437728 | 59242692 | 41064916 | 95.64 | 56.61 |
| BV_DON_2 | 54097388 | 51371390 | 35680940 | 95.64 | 56.6 |
| BV_DON_3 | 57518546 | 54609698 | 37527928 | 95.4 | 56.35 |
| V_DON_1 | 62287818 | 59125410 | 40976838 | 95.67 | 56.5 |
| V_DON_2 | 52402276 | 49746842 | 34503388 | 95.43 | 56.17 |
| V_DON_3 | 64445364 | 61091856 | 42387986 | 95.7 | 56.56 |
| AV_DON_1 | 61255186 | 58347738 | 40642426 | 95.47 | 57.39 |
| AV_DON_2 | 60389164 | 57479812 | 39421652 | 95.33 | 56.86 |
| AV_DON_3 | 73122202 | 69523602 | 48140124 | 95.29 | 57.46 |
| VG_DON_1 | 59849262 | 56952852 | 39491258 | 95.28 | 56.86 |
| VG_DON_2 | 54013620 | 51446398 | 35764512 | 95.48 | 56.83 |
| VG_DON_3 | 51336480 | 48843028 | 34259528 | 95.58 | 56.98 |
| BH_DON_1 | 62425430 | 61322786 | 40463486 | 95.4 | 57.09 |
| BH_DON_2 | 62051696 | 60996804 | 40952192 | 95.46 | 57.38 |
| BH_DON_3 | 63923754 | 62764420 | 42192308 | 95.56 | 57.07 |
| H_DON_1 | 58773888 | 57831790 | 37896776 | 95.56 | 55.33 |
| H_DON_2 | 80772370 | 79447036 | 53510954 | 95.47 | 56.05 |
| H_DON_3 | 67443164 | 66076598 | 44768672 | 95.61 | 55.86 |
| Total | 1108545336 | 1066220752 | 729645884 |  |  |

**Supplemental Table 1. RNA-seq statistics**
