# Supplementary material for: Comprehensive transcriptome analysis reveals distinct regulatory programs during vernalization and floral bud development of orchardgrass (Dactylis glomerata L.)
Source: BMC Plant Biol. 2017 Nov 22;17:216. doi: 10.1186/s12870-017-1170-8 (PMC5700690; doi:10.1186/s12870-017-1170-8)
Supplement: Supplementary file 6 — Statistics of annotation analysis of unigenes. (DOCX 16 kb) [file 12870_2017_1170_MOESM6_ESM.docx]

| **Annotation Database** | **Number of Unigenes** | **Percentage (%)** |
| --- | --- | --- |
|  |  |  |
| NR | 89838 | 33.24 |
| NT | 86173 | 31.88 |
| KO | 29180 | 10.79 |
| SwissProt | 65617 | 24.28 |
| PFAM | 70876 | 26.22 |
| GO | 72366 | 26.78 |
| KOG | 30874 | 11.42 |
| In all Databases | 13701 | 5.07 |
| In at least one Database | 133371 | 49.35 |
| Total Unigenes | 270221 | 100 |

**Supplemental Table 2. Statistics of annotation analysis of unigenes**
