# Supplementary material for: Comprehensive transcriptome analysis reveals distinct regulatory programs during vernalization and floral bud development of orchardgrass (Dactylis glomerata L.)
Source: BMC Plant Biol. 2017 Nov 22;17:216. doi: 10.1186/s12870-017-1170-8 (PMC5700690; doi:10.1186/s12870-017-1170-8)
Supplement: Supplementary file 11 — Identified flowering-related gene in orchardgrass. (DOCX 19 kb) [file 12870_2017_1170_MOESM11_ESM.docx]

|  |  |  |
| --- | --- | --- |
| Gene name | Unigene | Flowering pathway |
| WNK1 | c148831_g2 | Circadian clock |
| VRN1 | c147469_g1 | Vernalization |
| VIP2 | c146989_g2 | Vernalization |
| VIP1 | c137956_g2 | Vernalization |
| VIN3 | c135658_g1 | Vernalization |
| TOE3 | c138077_g1 | Photoperiod |
| TIC | c137328_g2 | Photoperiod |
| SWN | c121078_g3 | Vernalization |
| SVP | c197794_g1 | Vernalization |
| SUF4 | c137386_g1 | Vernalization |
| SPY | c140707_g1 | GA |
| SPL9 | c150483_g1 | Age |
| SPL5 | c44279_g1 | Age |
| SPL3 | c134262_g2 | Age |
| SPL | c131707_g1 | Age |
| SPA | c134073_g1 | Photoperiod |
| SOC | c116418_g2 | integrator |
| RGA | c101395_g1 | GA |
| REF6 | c129080_g4 | Vernalization |
| PRR7 | c152208_g1 | Circadian clock |
| PIE1 | c137377_g1 | Circadian clock |
| PHYE | c17166_g1 | Circadian clock |
| PHYB | c147487_g2 | Circadian clock |
| PHYA | c130359_g3 | Circadian clock |
| pEARLI | c129571_g1 | Vernalization |
| NF-YB2 | c116406_g3 | Photoperiod |
| NF-YB1 | c116940_g6 | Photoperiod |
| MSI4 | c202922_g1 | Autonomous |
| MBD9 | c139631_g1 | Photoperiod |
| MAF1 | c150421_g1 | Photoperiod |
| LWD1 | c107252_g1 | Circadian clock |
| LHY | c146679_g3 | Circadian clock |
| LHP1 | c143664_g4 | Vernalization |
| LD | c151781_g1 | Autonomous |
| HUA2 | c149571_g1 | Vernalization |
| GID1A | c128386_g2 | GA |
| GI | c151406_g1 | Photoperiod |
| GAI | c140748_g1 | GA |
| GA3 | c124218_g1 | GA |
| FY | c143631_g2 | Autonomous |
| FVE | c137063_g1 | Autonomous |
| FT | c142796_g3 | integrator |
| FRI | c149523_g1 | Vernalization |
| FPA | c149859_g1 | Autonomous |
| FLK | c142896_g1 | Autonomous |
| FLC | c147268_g1 | integrator |
| FIP2 | c148129_g1 | Vernalization |
| FIP1 | c147768_g1 | Vernalization |
| FES1 | c145565_g1 | Vernalization |
| FD | c128431_g1 | Photoperiod |
| FCA | c148856_g1 | Autonomous |
| ESD4 | c140400_g1 | Vernalization |
| EMF2 | c148889_g1 | Vernalization |
| ELF4 | c145757_g1 | Circadian clock |
| ELF3 | c149733_g2 | Circadian clock |
| CSTF77 | c147769_g1 | Vernalization |
| CSTF64 | c136423_g1 | Vernalization |
| CRY2 | c146958_g1 | Photoperiod |
| CRY1 | c137241_g1 | Photoperiod |
| COP1 | c122324_g1 | Photoperiod |
| COL4 | c121269_g2 | Photoperiod |
| COL3 | c127329_g2 | Photoperiod |
| COL2 | c160235_g1 | Circadian clock |
| COL1 | c151793_g1 | Circadian clock |
| CO | c138081_g1 | integrator |
| CLF | c150201_g1 | Vernalization |
| CDF3 | c143051_g1 | Photoperiod |
| CDF2 | c127155_g1 | Photoperiod |
| CCR2 | c135310_g1 | Autonomous |
| CCA1 | c146679_g3 | Photoperiod |
| CBF2 | c138744_g4 | Vernalization |
| CBF1 | c148965_g2 | Vernalization |
| ARR3 | c114208_g1 | Photoperiod |
| ARP4 | c135928_g1 | Photoperiod |
| APR4 | c151572_g1 | Vernalization |
| AGL19 | c108404_g1 | Vernalization |
| ABH1 | c147185_g1 | Photoperiod |

**Supplemental Table 3. Identified flowering-related gene in Orchardgrass**
